# The general law of plasma proteome alterations occurring in the lifetime of Chinese individuals reveals the importance of immunity

**DOI:** 10.18632/aging.204278

**Published:** 2022-09-07

**Authors:** Xiaolin Ni, Juan Jiao, Ze Yang, Zhaoping Wang, Nan Nan, Danni Gao, Liang Sun, Xiaoquan Zhu, Qi Zhou, Nan Zhang, Zhu Wu, Shenqi Zhang, Huiping Yuan

**Affiliations:** 1 The Key Laboratory of Geriatrics, Beijing Institute of Geriatrics, Institute of Geriatric Medicine, Chinese Academy of Medical Sciences, Beijing Hospital, National Center of Gerontology of National Health Commission, Beijing 100730, China; 2 University of Chinese Academy of Sciences, Beijing 100049, China; 3 State Key Laboratory of Stem Cell and Reproductive Biology, Institute of Zoology, Institute for Stem Cell and Regeneration, Chinese Academy of Sciences, Beijing 100101, China; 4 Department of Clinical Laboratory, The Seventh Medical Center, Chinese PLA General Hospital, Beijing 100700, China; 5 Department of Cardiology, Beijing Anzhen Hospital, Capital Medical University, Chaoyang, Beijing 100029, China; 6 Department of Joint and Sports Medicine, Zaozhuang Municipal Hospital Affiliated to Jining Medical University, Shandong 277100, China

**Keywords:** lifespan, proteomics, biological processes, immune system, aging

## Abstract

Background: Aging is characterized by a continuous loss of protein homeostasis. A closer examination of peripheral blood, which houses proteins from nearly all tissues and cells, helped identify several biomarkers and other aspects of aging biology. To further explore the general law of aging and identify key time nodes and associated aging biology, we collected 97 plasma samples from 253 healthy individuals aged 0-100 years without adverse outcomes to conduct nano-Ultra High Performance Liquid Chromatography-Mass Spectrometry/Mass Spectrometry (nano-UHPLC-MS/MS) and weighted gene co-expression network analysis (WGCNA).

Results: Through biological processes and key biological pathways identified in discrete age group modules, our analyses highlighted a strong correlation between alterations in the immune system and aging process. We also identified hub genes associated with distinct age groups that revealed alterations not only in protein expression but also in signaling cascade. Among them, hub genes from age groups of 0-20 years old and 71-100 years old are mostly involved in infectious diseases and the immune system. In addition, CDC5L and HMGB2 were the key transcription factors (TFs) regulating genes expression in people aged between 51-60 and 71-100 years of age. They were shown to not only be independent but also mutually regulate certain hub gene expressions.

Conclusions: This study reveals that the plasma proteome undergoes a complex alteration over the lifetime of a human. In this process, the immune system is crucial throughout the lifespan of a human being. However, the underlying mechanism(s) regulating differential protein expressions at distinct ages remains to be elucidated.

## INTRODUCTION

With a global increase in life expectancy and a simultaneous decrease in fertility, aging is of great concern in China and the rest of the world [1]. The aging process is marked with a complex multifactorial decline in organ function, as well as emergence of multiple chronic diseases, which may synergistically bring about death [2, 3]. Thus far, aging was mostly studied in simple model organisms [4].

Proteins can directly modulate cellular signaling. Aging is characterized by a continuous loss of protein homeostasis marked by massive imbalances in protein compositions [5]. Multiple studies demonstrated that the protein profile in cells, bodily fluids, and tissues undergo vast alterations with age. This can provide insight into the complex regulation of aging and related biological processes. In particular, evaluations of the peripheral blood, which houses proteins from nearly all cells and tissues of the body, helped in the identification of numerous aging biomarkers as well as aging biology. Therefore, the protein profile in peripheral blood is representative of age-related alterations in various cells and tissues [3]. Conversely, multiple reports confirmed that soluble factors from young mouse blood can effectively reverse aging in older mice [6]. Proteins are critical executive molecules that convey biological information from the genes to the rest of the cells. Often times, a protein forms a complex with other protein(s) to transmit information in a signaling cascade [7]. Weighted gene co-expression network analysis (WGCNA) is a highly efficient evaluation tool that highlights the association between genetic networks and heterogeneous traits. This is often used in the identification of physiological and pathological biomarkers [8].

Hence, we conducted plasma proteomics investigation in different age groups, ranging from infants to nonagenarians, using WGCNA, in order to identify age-related alterations in protein profile and its effects on biological systems and pathways. We also hoped to find some key proteins that are essential to the aging process and identify age- and longevity- specific genes.

## RESULTS

### Baseline characteristic

Between 2020–2021, we recruited 253 healthy individuals, aged 0–100 years, with no adverse outcomes (such as, cardiovascular diseases, neurodegenerative diseases, and cancers) from the Beijing Hospital. We collected plasma from ethylenediaminetetraacetic acid (EDTA)-treated blood obtained via vein puncture. Samples with poor plasma quality and incomplete basic information were excluded from the study. Next, we screened samples from each age group, which consisted of a compilation of 10 years (i.e., Age group 1: 0-10; Age group 2: 11-20; and so on). Finally, we selected 97 samples. Among them, 37 were males (38.14%) and 87 (89.69%) were Han Chinese (Table 1). These subjects were well matched in sex and ethnicity (Supplementary Table 1).

**Table 1:** Baseline characteristics of samples.

**Age**	**Mean±Std.**	**Num**	**Male, n(%)**	**Han, n(%)**	**State of health**
0-10	5.85±3.48	9	3(33.33)	6(66.67)	Yes
11-20	14.80±3.43	10	4(40.00)	9(90.00)	Yes
21-30	26.43±3.21	8	1(12.50)	7(87.50)	Yes
31-40	35.40±2.84	10	3(30.00)	10(100.00)	Yes
41-50	44.60±2.32	10	5(50.00)	9(90.00)	Yes
51-60	55.10±2.13	10	4(40.00)	9(90.00)	Yes
61-70	64.80±3.46	10	6(60.00)	10(100.00)	Yes
71-80	75.70±3.74	10	3(30.00)	10(100.00)	Yes
81-90	85.22±2.64	10	4(40.00)	10(100.00)	Yes
91-100	95.60±2.84	10	4(40.00)	7(70.00)	Yes
Total	-	97	37(38.14)	87(89.69)	Yes

Std, standard deviation; Num/n, number.

### Characterization of age-dependent protein expression

Mass spectrometry (MS)-based quantitative proteomics is a key technical method for protein identification and determination of the absolute or relative abundance of individual proteins [9]. In this study, we conducted MS quantitative proteomics based on Data-independent Acquisition (DIA). To quantify the proteomics data, peak strength of overall sample was calculated and normalized. Based on our analysis, the quantitative values distribution of the samples’ peptide segments achieved basically the same response strength (Supplementary Figure 2A). We examined protein quantification of all samples via LC-MS/MS to further validate our quantification data. Based on our analysis, DIA was very repetitive for large samples (Supplementary Figure 2B), suggesting that our proteomics technique was accurate and precise. In total, we identified 2682 age-related proteins (89.28%), out of the 3004 total proteins in plasma (false discovery rate [FDR]-corrected p≤0.01) (Supplementary Figure 2C). Following appropriate sample quality control (QC) and normalization procedures, we employed the Pearson correlation coefficients (PCC) to assess correlations among the 97 samples, based on proteomics data (2682 proteins). Apart from the A20-2 sample belonging to the Zhuang ethnic group, all PCCs between the technical replicates were relatively high, reaching mean of 0.97 (Supplementary Figure 2D).

### Construction of weighted gene co-expression network (WGCNA)

**Figure 1:**
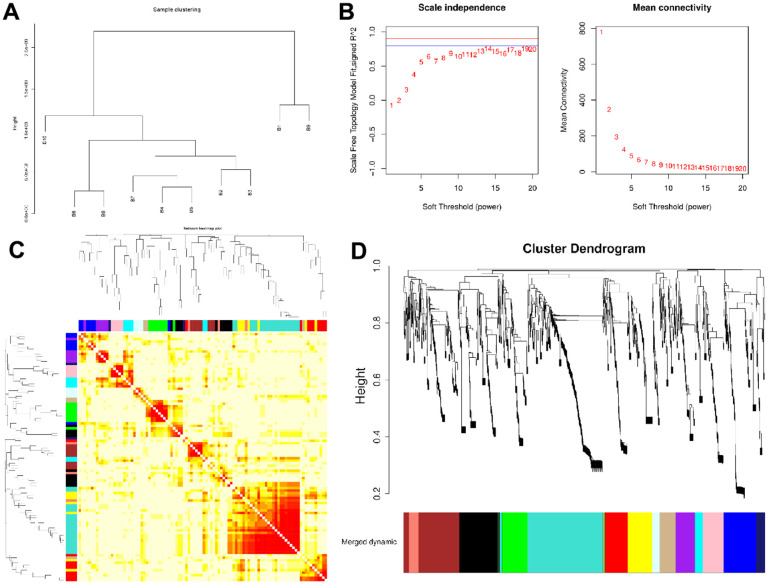
**Network construction of the weighted co-expressed genes and their associations with age.** (**A**) The Group hierarchical clustering tree. (**B**) Determination of soft-thresholding power. (**C**) The heatmap of genes correlation in modules. (**D**) Hierarchical clustering dendrograms (HCD) of modules. HCD using dynamic tree cut, depicting 14 plasma protein modules. Each branch of the HCD presents a single protein, and the colored bar below refers to its corresponding protein module. The HCD height is the distance between proteins.

WGCNA was conducted identify co-expression modules and generate co-expression networks. To conduct proteomics analysis, 96 samples were divided into 10 groups (B1-B10), with each group representing a compilation of 10 years old, following the removal of sample A20-2. Next, based on the expression level of all proteins, 10 sets of samples were clustered hierarchically. We observed no strong outliers (Figure 1A). Next, we employed adequate soft-threshold validation, with convergence toward scale-free topology and mean connectivity, to select 6 common values as power values (Figure 1B). The Dynamic Tree Cut algorithm provided the first set of modules and this was combined with associated modules (r > 0.7; Merged dynamic). Each module contained at least 50 genes (Figure 1C and Supplementary Figures 3, 4). 14 modules were identified according to age (Figure 1D).

### Correlation between modules and aging

**Figure 2:**
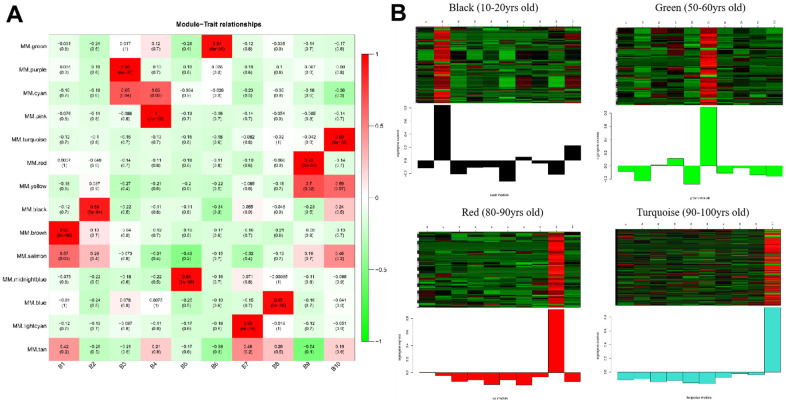
**Heatmaps of age-related modules and the clustering of some critical module eigengene.** (**A**) Heatmaps of the correlation between Eigengene and clinical traits of age. Each row corresponds to a module eigengene, and each column corresponds to an age group. Each cell contains the corresponding correlation and p-value. (**B**) The clustering of four module eigengenes (black, green, red and turquoise).

The gene co-expression in each module was summarized by Eigengene (i.e., the first component gene expression of each module). Next, we computed the relationship between each Eigengene and age group. As presented in Figure 2A and Supplementary Figure 5, the strongly correlated modules were different for each age group; for instance, the brown module represented 0-10 years old, the black module represented 11-20 years old, the purple module represented 21-30 years old, the pink module represented 31-40 years old, the midnight blue module represented 41-50 years old, the green module represented 51-60 years old, the lightcyan module represented 61-70 years old, the blue module represented 71-80 years old, the red module represented 81-90 years old, and the turquoise module represented 91-100 years old. In the meantime, the intramodular connectivity (K.in) and module correlation degree (MM) corresponding to each gene were computed and genes with high connectivity were assigned as potential hub genes with important functions. These results are presented in Supplementary Figures 6, 7.

### Functional enrichment analysis of critical modules

Next, we performed Gene Ontology (GO)-biological process (BP) and Kyoto Encyclopedia of Genes and Genomes (KEGG) enrichment analyses to elucidate the potential functional implications of proteins identified in the strongly correlated modules of each age group.

**Figure 3:**
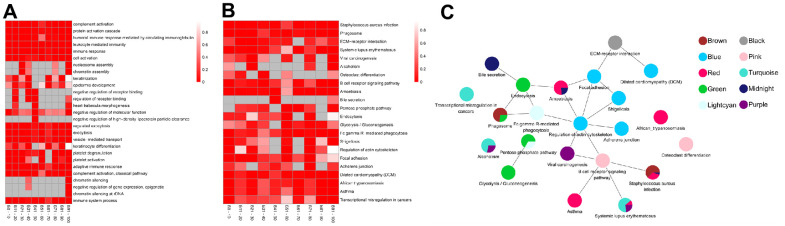
**The top enrichment of Gene Ontology (GO)-biological progress (BP) and Kyoto Encyclopedia of Genes and Genomes (KEGG) pathway in each age groups.** (**A**) The leading five enrichment data of GO-BP analysis. Differential genes (Genes) are distributed across multiple GO biological progress. Each row corresponds to the top five enriched pathways, and each column corresponds to an age group. The color represents P value and the smaller the P value, the color becomes darker (red). no enrichment to no color (White). There is no color (grey) without enrichment. (**B**) Top ten enrichment results of the KEGG pathway. Genes are involved in a wide range of KEGG. Each row corresponds to top ten enriched pathways, and each column corresponds to an age group. The color represents P value and the smaller the P value, the more obvious enrichment, the darker color (red), and no enrichment to no color (White). There is no color (grey) without enrichment. (**C**) The pathway crosstalk analysis of critical modules. The color of nodes denotes modules.

The top five BP-related GO terms from each age group demonstrated that the proteins were involved in complement activation, protein activation cascade, and immune response (GO:0006956, GO:0072376, GO:0002455) in the 0-10 years old (module brown); leukocyte mediated immunity (GO:0002443), immune response (GO:0006955), and cell activation (GO:0001775) in the 11-20 years old (module black); nucleosome or chromatin assembly (GO:0006334, GO:0031497) in the 21-30 years old (module purple); keratinization and epidermis development (GO:0031424, GO:0008544) in the 31-40 years old (module pink); regulation of receptor binding (GO:1900121, GO:1900120), heart trabecula morphogenesis (GO:0061384), negative regulation of molecular function or high-density lipoprotein particle clearance (GO:0044092, GO:0010987) in the 41-50 years old (module midnight blue); exocytosis (GO:0045055, GO:0006887) and vesicle-mediated transport (GO:0016192) in the 51-60 years old (module green); keratinization (GO:0031424, GO:0030216) in the 61-70 years old (module lightcyan); platelet degranulation or activation (GO:0002576, GO:0030168) in the 71-80 years old (module blue); protein activation cascade, complement activation, and immune response (GO:0072376, GO:0006956, GO:0002455, GO:0002250, GO:0006958) in the 81-90 years old (module red); chromatin silencing, epigenetic, and immune system process (GO:0006342, GO:0045814, GO:0000183, GO:0002376) in the 91-100 years old (module turquoise) (Figure 3A and Supplementary Table 3).

We also identified enriched pathways using KEGG enrichment analysis. Based on our analysis, infectious diseases (ko05150) were the most significantly enriched KEGG pathway, followed by transport and catabolism (ko04145) in the 0-10 years old (module brown). With the development of the body, signaling molecules and interaction (ko04512) with environmental information began to play key roles among the 11-20 years old (module black). The purple module, corresponding to the 21-30 years old, showed significant enrichment of pathways involved in human diseases, such as, immune diseases, cancers and substance dependence (ko05322, ko05203, ko05034). Subsequently, proteins were enriched in development and immune system (ko04380, ko04662) in the 31-40 years old (module pink), followed by infectious diseases (ko05146, ko05150) and digestive system (ko04976) in the 41-50 years old (module midnight blue). In the middle-aged and old stages [10] (51-60 years old, module green and 61-70 years old, module lightcyan), the proteins were significantly enriched in carbohydrate metabolism (ko00030, ko00010), transport, catabolism (ko04145, ko04144), and immune system (ko04666). These alterations implied that the cellular processes and metabolism were mobilized throughout the body. As the aging process continued, the proteins significantly enriched in cell motility and cellular community of cellular processes (ko04810, ko04510, ko04520), infectious diseases and cardiovascular diseases of human diseases (ko05131, ko05414) in the 71-80 years old (module blue). Next, proteins were enriched in immune and infectious diseases (ko05150, ko05143, ko05322, ko05146, ko05310) in the 81-90 years old (module red). Finally, in individuals over 90 years old, proteins were enriched in immune diseases, substance dependence, and cancers (ko05322, ko05034, ko05202; module turquoise; Figure 3B and Supplementary Table 4). Notably, the >90 age group was enriched in the same pathways as the 21-30 age group, however, the genes involved were different.

### The pathway crosstalk analysis of critical modules

Based on the overall analysis over an average lifespan, we performed pathway crosstalk analysis among the critical module in each age group. Notably, these modules were not independent, as they shared certain common pathways, for example, phagosome (ko04145), pentose phosphate pathway (ko00030), staphylococcus aureus infection (ko05150). All pathways in these modules formed two major clusters (Figure 3C). One cluster consisted of the pentose phosphate pathway and glycolysis/gluconeogenesis and involved the module green and lightcyan. The other cluster was more complex and formed connections with all critical modules. Interestingly, most of the signaling pathways in these clusters were related to immunity. Viral carcinogenesis, which was ranked as the topmost node (20 nodes) and most interactive, can serve critical roles in the progression of life. These results demonstrate that immunity is important throughout life.

### Identifying hub genes in the critical module

Furthermore, we generated a protein-protein interactions (PPI) network of proteins in the critical module of each age group (Table 2 and Supplementary Figure 8) to recognize chief hub genes associated with aging.

**Table 2:** The leading 10 expressed genes in the critical module of each age group.

**Module**	**Connectivity**	**Symbol**	**Description**
brown	91.829	FAP	fibroblast activation protein alpha
brown	90.954	IGLV4-3	immunoglobulin lambda variable 4-3
brown	87.384	DPEP2	dipeptidase 2
brown	87.381	COL11A2	collagen type XI alpha 2 chain
brown	87.381	TIE1	tyrosine kinase with immunoglobulin like and EGF like domains 1
brown	87.381	GPX1	glutathione peroxidase 1
brown	87.381	NOTCH2	notch receptor 2
brown	87.381	CLIC4	chloride intracellular channel 4
brown	86.847	HK1	hexokinase 1
brown	85.653	SPP2	secreted phosphoprotein 2
black	76.444	KRT14	keratin 14
black	74.998	CLIC1	chloride intracellular channel 1
black	69.874	KRT17	keratin 17
black	69.685	HPD	4-hydroxyphenylpyruvate dioxygenase
black	69.684	LAP3	leucine aminopeptidase 3
black	69.684	EEF2	eukaryotic translation elongation factor 2
black	69.683	FLT4	fms related receptor tyrosine kinase 4
black	69.683	LEP	leptin
black	68.911	COL6A1	collagen type VI alpha 1 chain
black	68.851	CTSS	cathepsin S
purple	55.565	H2BC	H2B clustered histone
purple	55.523	FLNA	filamin A
purple	51.639	PDIA3	protein disulfide isomerase family A member 3
purple	49.128	HSPD1	heat shock protein family D (Hsp60) member 1
purple	47.543	IGFBP5	insulin like growth factor binding protein 5
purple	46.371	FGL2	fibrinogen like 2
purple	45.153	ITGA6	integrin subunit alpha 6
purple	42.211	PGAM1	phosphoglycerate mutase 1
purple	39.598	ITGA2	integrin subunit alpha 2
purple	39.529	CHGA	chromogranin A
pink	72.830	KRTAP3-2	keratin associated protein 3-2
pink	72.830	LILR	leukocyte immunoglobulin like receptor
pink	72.830	KRTAP	keratin associated protein 9-8
pink	72.830	CCS	copper chaperone for superoxide dismutase
pink	72.830	KRTAP13-1	keratin associated protein 13-1
pink	72.788	KRTAP3-3	keratin associated protein 3-3
pink	72.673	KRTAP13-4	keratin associated protein 13-4
pink	72.348	KRT34	keratin 34
pink	72.022	KRTAP11-1	keratin associated protein 11-1
pink	69.581	KRT85	keratin 85
midnightblue	28.401	CTSH	cathepsin H
midnightblue	24.610	ENG	endoglin
midnightblue	23.752	SLCO1B3	solute carrier organic anion transporter family member 1B3
midnightblue	19.424	CHL1	cell adhesion molecule L1 like
midnightblue	17.789	LAMB1	laminin subunit beta 1
midnightblue	15.307	IGKV3	immunoglobulin kappa variable 3
midnightblue	12.843	PTPRF	protein tyrosine phosphatase receptor type F
midnightblue	11.532	RAB7A	RAB7A, member RAS oncogene family
midnightblue	11.048	APOC3	apolipoprotein C3
midnightblue	9.966	RNPEP	arginyl aminopeptidase
green	69.600	CORO1C	coronin 1C
green	66.963	NME2	NME/NM23 nucleoside diphosphate kinase 2
green	66.713	RAP1B	RAP1B, member of RAS oncogene family
green	65.605	RTN4	reticulon 4
green	65.236	ADH1	alcohol dehydrogenase 1
green	64.406	RAB6B	RAB6B, member RAS oncogene family
green	63.888	DPP3	dipeptidyl peptidase 3
green	60.382	CAP1	cyclase associated actin cytoskeleton regulatory protein 1
green	58.831	UBE2L5	ubiquitin conjugating enzyme E2 L5
green	58.221	LTA4H	leukotriene A4 hydrolase
lightcyan	28.009	VASP	vasodilator stimulated phosphoprotein
lightcyan	27.971	KRT82	keratin 82
lightcyan	22.800	WRAP73	WD repeat containing, antisense to TP73
lightcyan	22.141	GTF3C3	general transcription factor IIIC subunit 3
lightcyan	21.035	KRT36	keratin 36
lightcyan	19.421	RNH1	ribonuclease/angiogenin inhibitor 1
lightcyan	19.390	H6PD	hexose-6-phosphate dehydrogenase/glucose 1-dehydrogenase
lightcyan	19.028	CA6	carbonic anhydrase 6
lightcyan	16.826	GSTO1	glutathione S-transferase omega 1
lightcyan	16.259	C1orf56	chromosome 1 open reading frame 56
blue	93.696	MDH1	malate dehydrogenase 1
blue	91.610	IGF2R	insulin like growth factor 2 receptor
blue	90.917	COL1A2	collagen type I alpha 2 chain
blue	90.890	APP	amyloid beta precursor protein
blue	90.882	PAFAH1B2	platelet activating factor acetylhydrolase 1b catalytic subunit 2
blue	90.880	PSMA1	proteasome 20S subunit alpha 1
blue	90.880	AC018523.2	novel protein
blue	90.879	CCL5	C-C motif chemokine ligand 5
blue	90.879	ANGPTL6	angiopoietin like 6
blue	90.878	CYCS	cytochrome c, somatic
red	72.930	ECI1	enoyl-CoA delta isomerase 1
red	72.929	PIP	prolactin induced protein
red	71.924	EP400	E1A binding protein p400
red	71.675	PRR16	proline rich 16
red	71.605	MYZAP	myocardial zonula adherens protein
red	71.605	GCOM1	GRINL1A complex locus 1
red	65.762	AMY	amylase alpha
red	62.372	KPRP	keratinocyte proline rich protein
red	59.745	KRT9	keratin 9
red	58.541	IGKV2D-24	immunoglobulin kappa variable 2D-24 (non-functional)
turquoise	202.101	HMBS	hydroxymethylbilane synthase
turquoise	202.101	GKN1	gastrokine 1
turquoise	202.100	H3	H3
turquoise	202.100	TPT1	tumor protein, translationally-controlled 1
turquoise	202.100	LGALS7B	galectin 7B
turquoise	202.100	SVEP1	sushi, von Willebrand factor type A, EGF and pentraxin domain containing 1
turquoise	202.100	PGM2	phosphoglucomutase 2
turquoise	202.100	SSC5D	scavenger receptor cysteine rich family member with 5 domains
turquoise	202.100	SPINK1	serine peptidase inhibitor Kazal type 1
turquoise	202.099	MYL1	myosin light chain 1

The all-connective genes in the ten modules were individually uploaded to STRING for PPI evaluation (Supplementary Figure 8). In all, the node scores ranged from 0.400 to 0.999. Using the PPI data, we were also able to establish the co-expression coefficient of different proteins. Based on the high connectivity of genes in each module, the elevated combined scores (CS; value > 0.9) and elevated co-expression coefficients (CEC; value > 0.8) suggested that protein-protein interactions dictate spatial protein positioning and gene expression within cells. We further analyzed the genes in relation to our previously identified hub genes in each age group. The molecular functions of all candidate hub genes are listed in Table 2. And, elevated CS (value > 0.9), but reduced CEC (value = 0) suggests that the protein-protein interaction not only regulates gene expression, but also signaling cascade (Supplementary Table 5).

**Figure 4:**
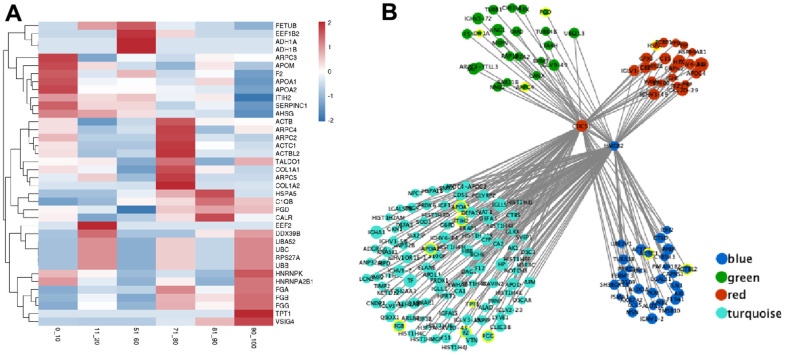
**The expression of hub genes and transcription factor (TF) regulatory network in the critical module.** (**A**) The expression heatmap of hub gene. (**B**) The interaction regulatory network of TF and target genes. The core circles are two transcription factors (CDC5L and HMGB2). The surrounding circles represent their target genes, the color represents the source module, the circle size represents the expression, and the yellow circle represents the hub gene.

Based on our results, there were forty hub genes in the module brown, black, green, blue, red, and turquoise with elevated CS combined scores (value > 0.9) and elevated CEC (value > 0.8) (Table 3). The proteins in these modules represented strong correlation with the corresponding age groups (Figure 4A). Interestingly, hub genes from age groups of 0-20 years old and 71-100 years old, namely, ARPC2, UBB, FGB, and FGG, are mostly involved in infectious diseases and the immune system. In contrast, the hub genes from the age groups of 51-60 years old, namely, ADH1A, PGD, and TALDO1, are mainly associated with carbohydrate metabolism (Table 3 and Supplementary Table 6).

**Table 3:** The protein–protein interaction (PPI) analysis of hub genes in different modules.

**Age groups**	**Module**	**Node1**	**Node2**	**Node1 connectivity**	**Node2 connectivity**	**Coexpression**	**Combined score**
0-10 years old	brown	ARPC2	ARPC5	16.766	18.201	0.946	0.999
11-20 years old	black	DDX39B	HNRNPA2B1	49.029	66.056	0.950	0.970
		DDX39B	HNRNPK	49.029	64.876	0.951	0.960
		EEF2	UBA52	69.684	54.320	0.924	0.999
		HNRNPA2B1	HNRNPK	66.056	64.876	0.953	0.998
		RPS27A	UBA52	54.320	54.320	0.935	0.999
		UBB	UBC	54.320	54.320	0.858	0.998
51-60 years old	green	ADH1A	ADH1B	65.236	65.236	0.828	0.982
		ARPC3	ARPC4	22.205	24.819	0.999	0.999
		PGD	TALDO1	8.834	4.664	0.863	0.990
71-80 years old	blue	ACTB	ACTC1	25.966	57.476	0.948	0.984
		ACTB	ACTBL2	25.966	52.264	0.946	0.946
		ACTBL2	ACTC1	52.264	57.476	0.939	0.939
		COL1A1	COL1A2	16.749	90.917	0.991	0.999
81-90 years old	red	CALR	HSPA5	14.716	13.575	0.881	0.998
91-100 years old	turquoise	AHSG	APOA1	72.648	48.668	0.918	0.996
		AHSG	FGA	72.648	45.072	0.835	0.989
		AHSG	F2	72.648	50.781	0.959	0.973
		AHSG	FGG	72.648	48.344	0.864	0.991
		AHSG	ITIH2	72.648	132.353	0.841	0.990
		AHSG	SERPINC1	72.648	58.881	0.807	0.987
		AHSG	APOA2	72.648	48.962	0.918	0.995
		APOA1	APOA2	48.668	48.962	0.902	0.999
		APOM	FGB	28.989	45.765	0.919	0.931
		C1QB	VSIG4	28.621	11.071	0.955	0.967
		EEF1B2	TPT1	46.912	202.100	0.886	0.973
		F2	FGA	50.781	45.072	0.804	0.999
		F2	FGG	50.781	48.344	0.811	0.998
		F2	SERPINC1	50.781	58.881	0.815	0.999
		FETUB	ITIH2	19.827	132.353	0.916	0.939
		FGA	FGB	45.072	45.765	0.992	0.999
		FGA	ITIH2	45.072	132.353	0.845	0.985
		FGA	SERPINC1	45.072	58.881	0.925	0.996
		FGA	FGG	45.072	48.344	0.993	0.999
		FGB	SERPINC1	45.765	58.881	0.838	0.991
		FGB	FGG	45.765	48.344	0.992	0.999
		FGG	ITIH2	48.344	132.353	0.886	0.989
		FGG	SERPINC1	48.344	58.881	0.907	0.995
		ITIH2	SERPINC1	132.353	58.881	0.922	0.994

### Construction of the transcription factor (TF) regulatory network

We further investigated the molecular mechanisms that regulate hub genes. We employed the Network Analyst tool to predict TFs that regulate expression of the forty hub genes. Based on our analysis, the TFs CDC5L and HMGB2 modulated the expressions of ADH1A, ARPC4, PGD, HSPA5, APOA1, ITIH2, APOA2, TPT1, FGB, F2, FGG, ACTC1, and ACTBL2 genes (Figure 4B) in the 51-60 years old and 71-100 age groups (modules blue, green, red, and turquoise). In addition, PCC analysis revealed that CDC5L was positively correlated with ADH1A, ACTC1, APOA1, ITIH2, and FGG expressions, whereas, HMGB2 was positively correlated with ARPC4, PGD, HSPA5, APOA1, ITIH2, APOA2, TPT1, FGB, F2, and ACTBL2 expressions.

## DISCUSSION

Using extensive plasma proteome analysis, we identified several undulating protein alterations that occur at different stages of a human lifetime (Supplementary Figure 5). We further aggregated these Genes into clusters within distinct age groups or modules. In addition, we analyzed the biological processes and key biological pathways of each age group/module, and revealed some interesting patterns associated with aging. Moreover, we demonstrated that the immune system plays a crucial role in all age groups.

Based on our analysis, from birth to ten years of age, proteins were mainly enriched in complement activation, protein activation cascade, and immune response (GO:0006956, GO:0072376, GO:0002455, GO:0006958, GO:0006959). It is well known that complement is a key component of the innate immune reference and it forms a gateway to adaptive immune responses [11]. In particular, invasive bacterial infections in childhood are strongly related to significant morbidity and death [12], indicating that the body's response to the external environment must be the main physiological activity in young children. This is in line with the characteristics of infants and young children who adapt to changes in the external environment (ko05150) and simultaneously carry out their own material transport and catabolism (ko04145). With strong adaptation to the external environment, the immune response remains the main biological process and exerts its action via signaling molecules and interaction (ko04512) with environmental information in 11-20 years old.

In ages 21-30 years old, proteins were mostly enriched in nucleosome assembly, chromatin assembly, and nucleosome organization (GO:0006334, GO:0031497, GO:0034728), involving human diseases like immune diseases, cancers, and substance dependence (ko05322, ko05203, ko05034). Prior reports revealed that substance dependence is a chronic relapsing brain disorder caused by alterations in synaptic resilience and neuronal activities. It is also a common cause of adolescent morbidity and mortality in the United States [13, 14].

The National Cancer Institute defines cancer amongst adolescents and young adults (AYAs) as a unique form of cancer affecting 15 to 39 years old. Multiple evidences suggest that AYA tumors are molecularly distinct, relative to cancers in other age groups [15]. Our results also revealed that although the proteins in the 21-30 and 91-100 age groups enriched in the same disease pathways, the genes involved were vastly different. For instance, H2B clustered histone was more prevalent in 21-30 years old, whereas H3 clustered histone was more active in the 91-100 years old. The active biological processes in the 91-100 years old age group involved chromatin silencing and negative regulation of gene expression (GO:0006342, GO:0045814), which were also different from the 21-30 years old. However, the specific mechanisms that make people vulnerable to these diseases need to be further explored.

In the 31-40 years old, keratinization and epidermis development (GO:0031424, GO:0008544) were important factors in maintaining immunity. The epidermis is the outer layer of the skin. It is in direct contact with the external environment, it protects our body from dehydration, and prevents xenobiotic penetration or infection of the organism. It is also representation of our health and of what is going on inside the body [16]. In our study, once the age reached 40 years old, organs and body molecules began to change gradually, such as, heart trabecula morphogenesis, high-density lipoprotein (HDL) particle clearance (GO:0061384, GO:0010987), indicating the gradual increase of internal regulation in the organism. In the middle-aged and old stages [10] (51-70 years old), proteins were mostly enriched in the carbohydrate metabolism, transport, and catabolism processes (ko00030, ko04145, ko04144, ko00010). Recent studies revealed that a low-protein and high-carbohydrate diet strongly regulates longevity and metabolic health [17]. Geometric Framework examinations on insects and mice demonstrated that diets low in protein and high in carbohydrates produce the longest lifespans in ad libitum-fed animals [18]. In particular, hub genes, such as, ADH1A, PGD, and TALDO1, in the module green (51-60 years old) are also mainly associated with carbohydrate metabolism (Table 3). ADH1A (alcohol dehydrogenase 1A) catalyze alcohol oxidation to aldehydes. Zahid et al. suggested that higher ADH1A expression is associated with good survival and a less aggressive disease state in hepatocellular carcinoma patients [19]. 6-phosphogluconate dehydrogenase (PGD) is a cytosolic enzyme involved in carbohydrate metabolism [20]. A review reported that 6PGD deficiency reduced lipogenesis and RNA production while increasing ROS expression in cancer cells, thus suppressing cell proliferation and tumour growth [21]. TALDO1 (Transaldolase 1) is a chief enzyme belonging to the nonoxidative pentose phosphate pathway that provides ribose-5-phosphate for nucleic acid generation and NADPH for lipid biosynthesis. TALDO is clinically related to carbohydrate metabolism, and involves multiple organ systems, such as, breast, liver, pancreas, kidney and heart [22]. Our results demonstrated that in 51-60 years old, there is a strong relationship between these genes (ADH1A, PGD and TALDO1), carbohydrate metabolism, and health in general. Thus, we speculated that the middle- and old ages may be a critical period for the metabolism-mediated regulation of longevity in humans. With the development of the body's aging process (71-90 years old), the occurrence risk of immune, infectious, and cardiovascular diseases increases. Multiple studies of cardiovascular diseases revealed that the adaptive immune response plays a crucial role in a wide range of cardiovascular disorders [23]. With increasing age, immunologic activities decline in a process called immunosenescence. This decline is immune activity causes higher incidences and severity of infectious and cardiovascular diseases in elderly persons [23, 24]. Therefore, although the biological process (GO:0072376, GO:0006956, GO:0002455, GO:0002250, GO:0006958) of proteins enrichment in the 81-90 years old was similar to that of infants and young children, it may be caused by immunosenescence, not immature immune cells, according to previous researches and literature reports.

TFs are special protein molecules that can bind to specific DNA sequences and influence gene expression [25]. Here, for the first time, we demonstrated that CDC5L and HMGB2 are key TFs regulated genes in the age groups 51-60 and 71-100 years. Studies revealed that the cell division cycle 5-like (CDC5L) protein modulates the G2/M transition and participates in the catalytic stage of pre-messenger RNA (mRNA) splicing and DNA damage repair [26]. High-mobility group box (HMGB)2 protein is involved in DNA replication, repair, transcription, differentiation, proliferation, cell signaling, inflammation, and tumor migration [27]. In our study, CDC5L and HMGB2 were shown to not only be independent but also mutually regulate certain hub gene expressions (Figure 4B), namely, it regulated the pentose phosphate pathway (ko00030) and glycolysis/gluconeogenesis (ko00010) in carbohydrate metabolism, dilated cardiomyopathy (DCM) (ko05414) associated with cardiovascular diseases, as well as complement and coagulation cascades (ko04610) and staphylococcus aureus infection (ko05150) in the immune system (Supplementary Table 4). CDC5L and HMGB2 may play different roles at different ages.

Although we found some interesting regular patterns with ageing for the first time, especially the immune system throughout the whole life phase. Additionally, large-scale population validation and experimental validation in cell or animal models are still needed. Meanwhile, a special-purpose questionnaire survey on the subjects in terms of lifestyle which may alter plasma proteomic expression levels will also need to conduct in the future.

## CONCLUSION

Here, using WGCNA, we identified clusters of proteins distributed across distinct modules. The results from this study demonstrated that the plasma proteome undergoes a complicated alteration over the lifetime of a human being. In-depth analyses of age-related proteins revealed significant biological processes and key biological pathways within each age group/module, and identified some interesting regular patterns associated with aging, namely the immune system, which is crucial throughout the lifespan of a human being. We also identified distinct hub genes corresponding to different ages. Among them, hub genes are mostly involved in carbohydrate metabolism at the age groups of 51-60 years old which may be a critical period for the metabolism-mediated regulation of longevity in humans. In addition, we highlighted CDC5L and HMGB2 as key TFs regulating gene expression in the 51-60 and 71-100 years old people. However, the specific mechanisms whereby genes modulate target biological processes at different ages need to be further explored.

## MATERIALS AND METHODS

### Subjects

Samples in this study were selected from 253 healthy individuals, aged 0–100 years, without infectious diseases and adverse outcomes like cardiovascular diseases, neurodegenerative diseases, and cancers. We collected plasma from EDTA-treated blood obtained via vein puncture. According to the inclusion / exclusion criteria (inclusion criteria: 0-100 years old, healthy individuals with complete basic information; exclusion criteria: 0-100 years old, with infection or major chronic diseases, or with incomplete basic information), 153 individuals were screened out. Finally, we selected 97 samples (37 males and 60 females, 87 Han Chinese and 10 other Ethnic Groups) according to the principle of 10-year-old group (i.e., Age group 1: 0-10; Age group 2: 11-20; and so on), equal distribution of sample number and gender (Table 1). Subjects in the ten groups were well matched in sex and ethnic by chi-square test (Supplementary Table 1). We received informed consent from all subjects and their guardians, retrieved detailed information about this research and any risks associated with it before subject recruitment. Our protocols followed the Declaration of Helsinki and received approval from the ethical board at Beijing Hospital, Ministry of Health (2019BJYYEC-118-02).

### Sample pretreatment for plasma proteome profiling using DIA MS

The plasma sample (20 μL for serum sample) was diluted with 1% SDS in 8 M urea buffer 40 times and allowed to lyse on ice for 30 min, while vortexing the tube every 10 min. The soluble protein lysates were then centrifuged at 12000 g for 20 min at 4° C and the accumulated protein quantified with a BCA protein assay (Supplementary Figure 1 and Supplementary Table 2). Next, 100 μg of protein was placed in a fresh Eppendorf tube with 8 M urea at a total volume to 100 μL. 2 μL of 0.5 M TCEP was then introduced and the sample maintained at 37° C for 1 h, before addition of 4 μL of 1 M iodoacetamide and maintenance in the dark at room temperature (RT) for 49 min. Following this, 5 volumes of -20° C pre-chilled acetone was employed for overnight (ON) protein precipitation at -20° C. The precipitated protein was then rinsed with pre-chilled 90% acetone aqueous solution two times before re-suspension in 100 μL 100 mM TEAB. This was followed by the addition of Sequence grade modified trypsin (Promega, Madison, WI; ratio 1:50, enzyme: protein, weight: weight) for ON protein digestion at 37° C. The peptide mixture was then desalted using C18 ZipTip, measured with Pierce™ Quantitative Colorimetric Peptide Assay (23275), and lyophilized using SpeedVac.

### High PH reverse phase separation

The peptide mixture was re-suspended in 20mM ammonium formate in water, pH brought up to 10.0 with ammonium hydroxide. Next, it underwent fractionation via high pH separation using Ultimate 3000 system (Thermo Fisher Scientific, MA, USA) attached to a reverse phase column (XBridge C18 column, 4.6mm x 250 mm, 5μm, (Waters Corporation, MA, USA). It employed a linear gradient from 5-45% in 40 min of 20mM ammonium formate in 80% ACN, pH brought up to 10.0 with ammonium hydroxide. Column re-equilibration was done for 15 min to bring it back to the starting condition. The column flow rate (CFR) and temperature (CT) remained at 1mL/min and 30° C, respectively. We collected a total of ten fractions, each of which was vacuum-concentrated for subsequent analysis (Guangzhou Gene Denovo Bio-Tech Co., Ltd., China).

### DDA: Nano-HPLC-MS/MS analysis

The peptides were re-suspended in 30 μL 0.1% formic acid in water and assessed via on-line nanospray LC-MS/MS on an Orbitrap Fusion Lumos coupled to EASY-nLC 1200 system (Thermo Fisher Scientific, MA, USA). 3 μL peptide was introduced to the analytical column (Acclaim PepMap C18, 75 μm x 25 cm) and peptide separation was done with a 120-min gradient spanning 5-35% concentration of 0.1% formic acid in CAN. The CFR was adjusted to 200 nL/min and the CT to 40° C. The electrospray voltage of 2 kV versus the MS inlet was employed.

MS was carried out in data dependent acquisition mode, which automatically changed between MS and MS/MS modes. The adjustment criteria were as follows: (1) MS: scan range (m/z) =350–1200; resolution=120,000; AGC target=400000; maximum injection time=50 ms; Filter Dynamic Exclusion: exclusion duration=30s; (2) HCD-MS/MS: resolution=15,000; AGC target=50000; maximum injection time=35 ms; collision energy=32.

### Database search

Raw DDA data processing and analysis was done with Spectronaut X (Biognosys AG, Switzerland), using default settings, to develop the initial search list. The search criteria included homo sapiens and trypsin as the digestion enzyme. Carbamidomethyl (C) was assigned as the fixed alteration. Oxidation (M) was assigned as the variable alteration. The Q value (FDR) threshold for precursor and protein levels was set to 1%.

### DIA: Nano-HPLC-MS/MS analysis

The peptides were re-suspended in 30 μL of 0.1% formic acid in water and assessed as described above. The parameter adjustments were similar to the ones described above, except for a few alterations as is listed below: (1) MS: AGC target=1e6; (2) HCD-MS/MS: resolution=30,000; AGC target=1e6; collision energy=32; stepped CE=5%. (3) DIA was conducted with variable Isolation window, and each window overlapped 1 m/z, and the window quantity was 60.

### Data analysis

Data preparation and analysis were done as described above. We chose dynamic iRT as the retention time estimation type. Data retrieval via Spectronaut Pulsar [28] employed a large-scale mass calibration that used iRT calibration and gradient stability to generate an ideal dynamic extraction window. Precursor and protein q value (FDR) threshold was set to 1%. We also selected “mutated” decoy generation, which applied an arbitrary quantity of random numbers of AA position swamps (min=2, max=length/2). Subsequently, we quantified all identified precursors that passed the adjusted filters. The mean top 3 filtered peptides which passed the 1% Q value threshold were entered into the calculation of the key group quantities. Missing values are not filtered or filled in our study. The specific results of quantitative peptides and expression differential analysis, please refer to Supplementary Figure 2.

### Relationship analysis of samples

### Correlation analysis (CA) of replicas

CA was conducted via R. This analysis produces a correlation coefficient (CC), which enables users to evaluate the reliability and operational stability of two parallel experiments. Experiment repeatability enhances the closer CC is to 1.

### WGCNA generation and age-related modules identification

### Network construction

Co-expression networks were generated with the WGCNA (v1.47) package in R [29]. Following the elimination of irrelevant genes, the DEG values were entered into WGCNA to generate co-expression modules, based on the blockwise automatic network generation tool. Modules were thus generated with mostly default settings, except for a few adjustments as follows: power: 6; TOM Type: unsigned; merge Cut Height: 0.7; and min Module Size: 50. Subsequently, genes placed in clusters in 14 associated modules.

### Module and gene selection

To identify clinically relevant modules, we employed module eigengenes to compute the CC of samples or sample traits. Intramodular connectivity (K.in) and module correlation degree (MM) of each gene were computed with the R package of WGCNA and genes with relatively high connectivity were identified as potential hub genes carrying clinically relevant functions. PCC between each gene and corresponding trait information was computed for all modules and gene significance value (GS) was measured for all genes. The generated networks were visualized in Cytoscape_3.3.0 [30].

### Functional analysis of module genes

We also performed GO and KEGG pathway enrichment analysis on genes from all modules to discern the biological activity of the relevant genes in each module.

### Functional analysis of module genes

Gene Ontology (GO) [31] is an internationally recognized gene function stratification system. It provides the most recent definitions and properties of any genes and their products in an organism. GO examines three major categories: molecular function (MF), cellular component (CeCom), and biological process (BP). Its smallest unit is GO-term, which refers to a specific type of ontology. GO enrichment analysis enables us to determine which GO terms are markedly enriched in a given module, relative to the genome background. Using this information, one can screen for module genes that represent certain BP. We first mapped all module genes to the GO terms in the Gene Ontology database (http://www.geneontology.org/). Next, we calculated gene numbers for every term, before screening for markedly enriched GO terms, relative to background genome, as evidenced by the hypergeometric test. P-value was determined as follows


$$P=1-\sum_{i=0}^{m-1}\frac{\left(\begin{array}{c} M\\ i \end{array})(\begin{array}{c} N-M\\ n-i \end{array}\right)}{\left(\begin{array}{c} N\\ n \end{array}\right)}$$

Here, N represents GO annotation-associated gene quantity; n denotes gene quantity in the module N; M represents the total quantity of specific GO term-related genes; m denotes gene quantity in the module M. The computed p-value underwent FDR Correction, considering FDR ≤ 0.05 as the cut-off point. GO terms meeting these criteria were assigned as markedly enriched GO terms in module genes.

### Pathway enrichment analysis (PEA)

Protein-protein interactions are crucial regulators of biological functions. PEA is an excellent tool for elucidating the biological roles of genes in cells. KEGG is a commonly used public pathway-associated database [32]. Using PEA, we recognized essential signaling pathways associated with the Genes in the ten examined modules, relative to a genome background. The formula used to perform PEA was the same as GO terms, only instead of entering genes associated with GO terms, we entered genes associated with the KEGG annotation.

### Protein-protein interaction (PPI) network generation using select modules and identified hub genes

PPI network was generated via STRING (version 10.0; available online: http://string-db.org) [33], which plotted genes as nodes and interaction as lines, and it was visualized via the Cytoscape software [30], which displayed a core and hub gene biological interaction. The generated PPI network represented a database of known and predicted protein interactions, and only interactions with a combined score >0.9 were marked as statistically significant.

### Protein advanced functional annotation

### The protein domain analysis (Pfam annotation)

Pfam (Protein families database of alignments and hidden Markov models) is a database that includes protein structural information, based on the widely used multiple sequence alignment and hidden markov model (HMM). Protein domain prediction employed the Pfam_scan [34] program developed by sanger. The protein sequence was compared with the Pfam database to obtain relevant annotation information of protein structures.

### TF analysis

TFs regulate gene expression and are critical for development, cellular function, and disease response. The predicted protein sequences were compared with the TF database (plantTFDB [35]/animalTFDB [36]) using hmmscan.

### Subcellular localization analysis

Protein activities are highly reliant upon their subcellular localizations. Hence, predicting protein localization directly from protein sequences can be highly beneficial in inferring protein functions. The software WoLFPSort [37] was used to predict the subcellular location of proteins and study their functions.

### Availability of data and material

The datasets generated during and/or analyzed during the current study are available from the corresponding author on reasonable request.

## Supplementary Material

Supplementary Figures

Supplementary Tables 1-3

Supplementary Tables 4 and 5

Supplementary Table 6
