# Supplementary material for: The general law of plasma proteome alterations occurring in the lifetime of Chinese individuals reveals the importance of immunity
Source: Aging (Albany NY). 2022 Sep 7;14(17):7065–92. doi: 10.18632/aging.204278 (PMC9512505; doi:10.18632/aging.204278)
Supplement: Supplementary Figures [file aging-14-204278-s001.pdf]

SUPPLEMENTARY FIGURES

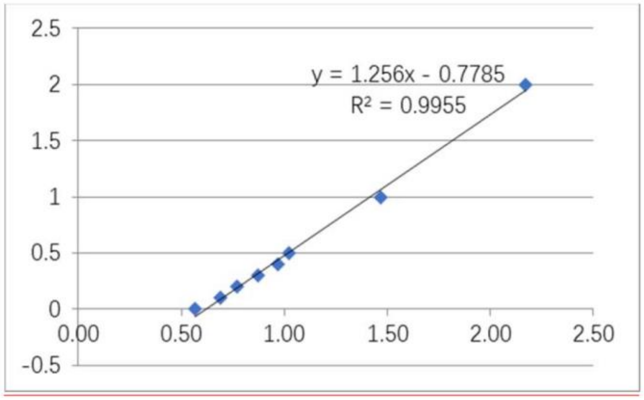

Supplementary Figure 1. The quantitative standard curve of BCA.

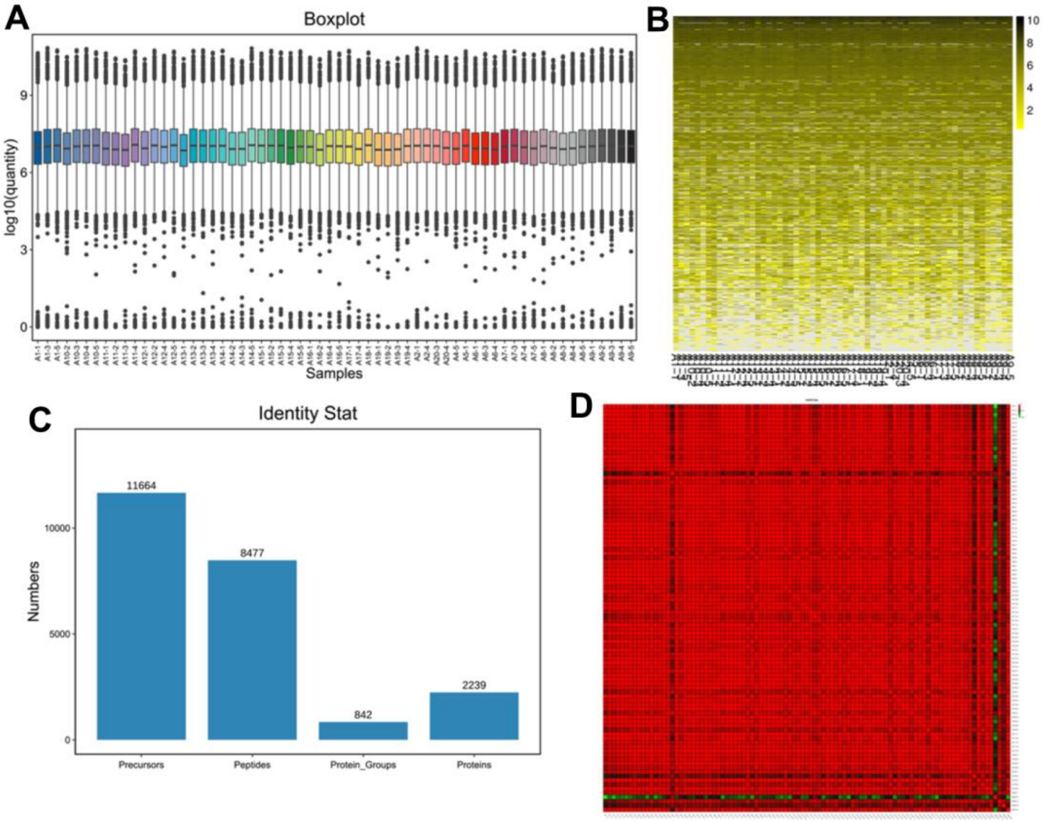

Supplementary Figure 2. The analysis of sample quality and sample relationship. (**A**) The violin plots. The data density of all proteins anywhere in each age group and protein abundance expression in samples or inter groups are shown for visualization. (**B**) The heatmap of quantitative protein in samples. (**C**) Statistical results of protein and peptide segment identification. (**D**) The correlation heatmap of samples. The Pearson correlation between two samples computed to analyze the difference between repeating samples and samples between groups.

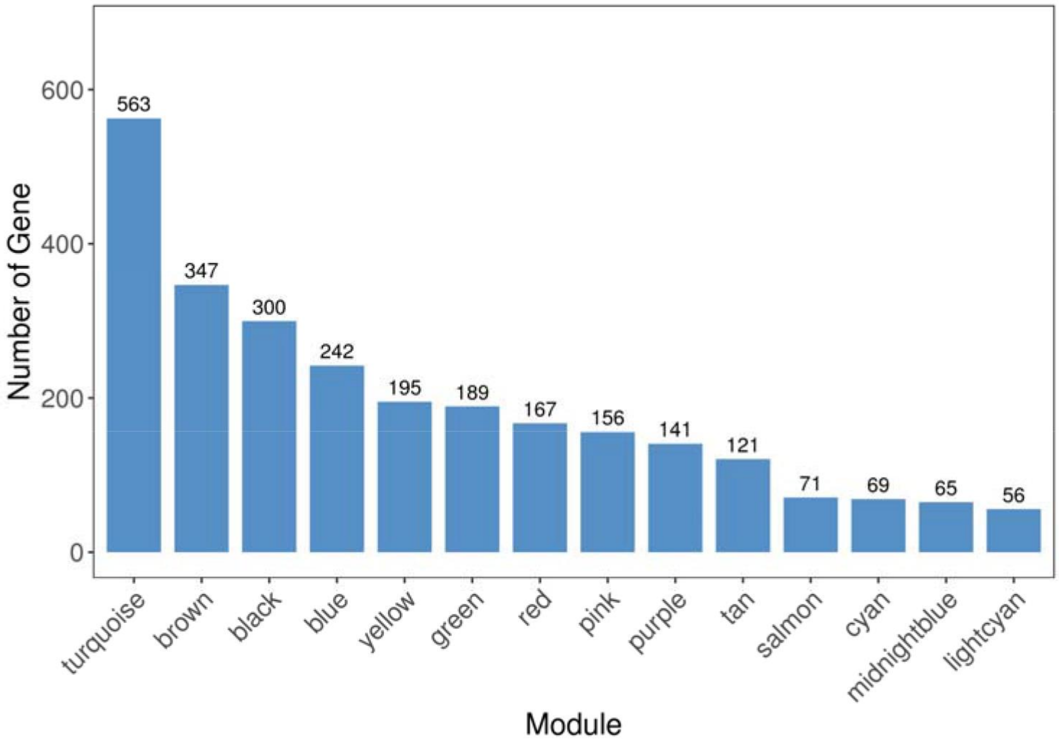

Supplementary Figure 3. The number of genes in each module.

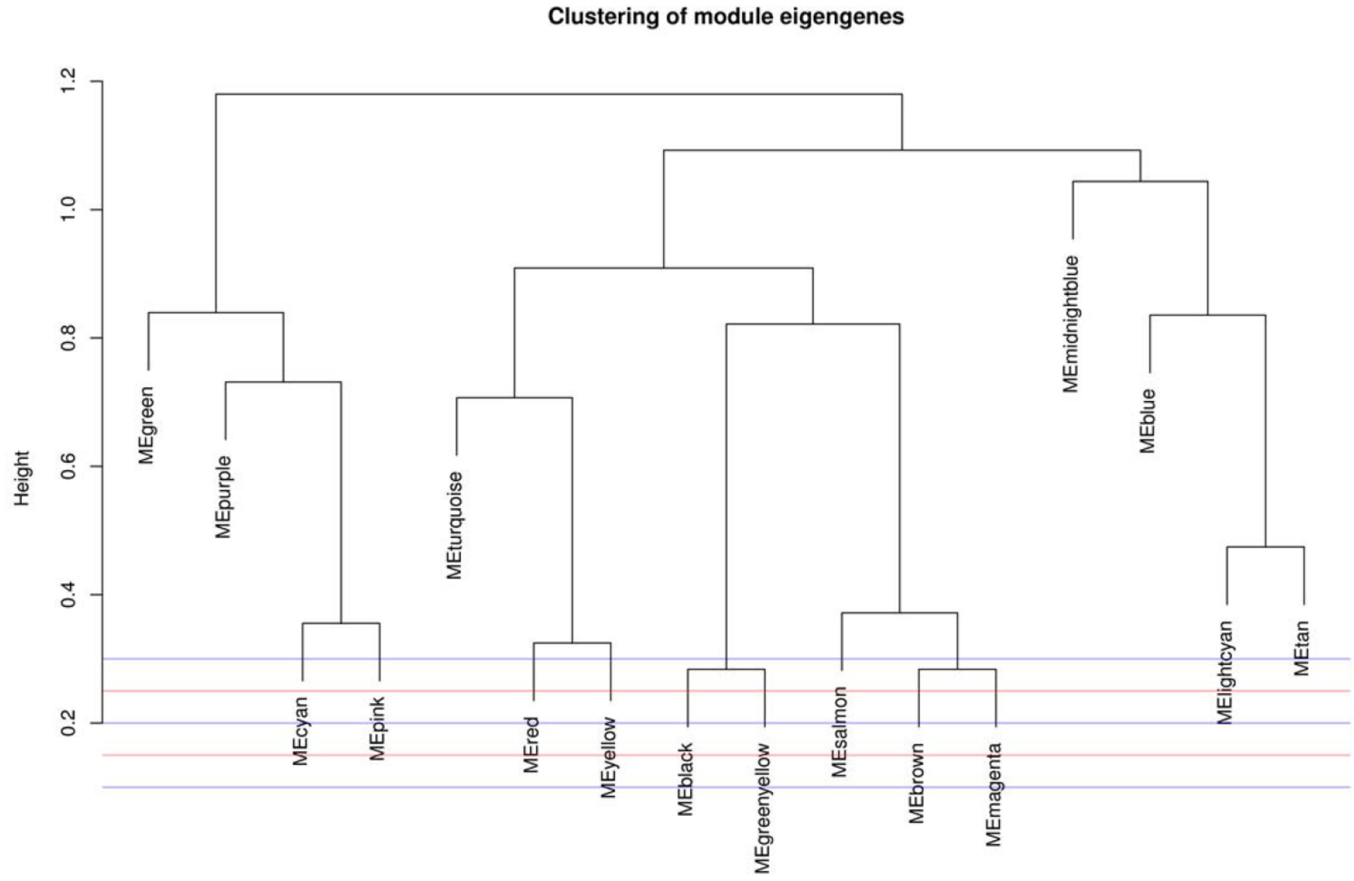

Supplementary Figure 4. The clustering of module eigengene.

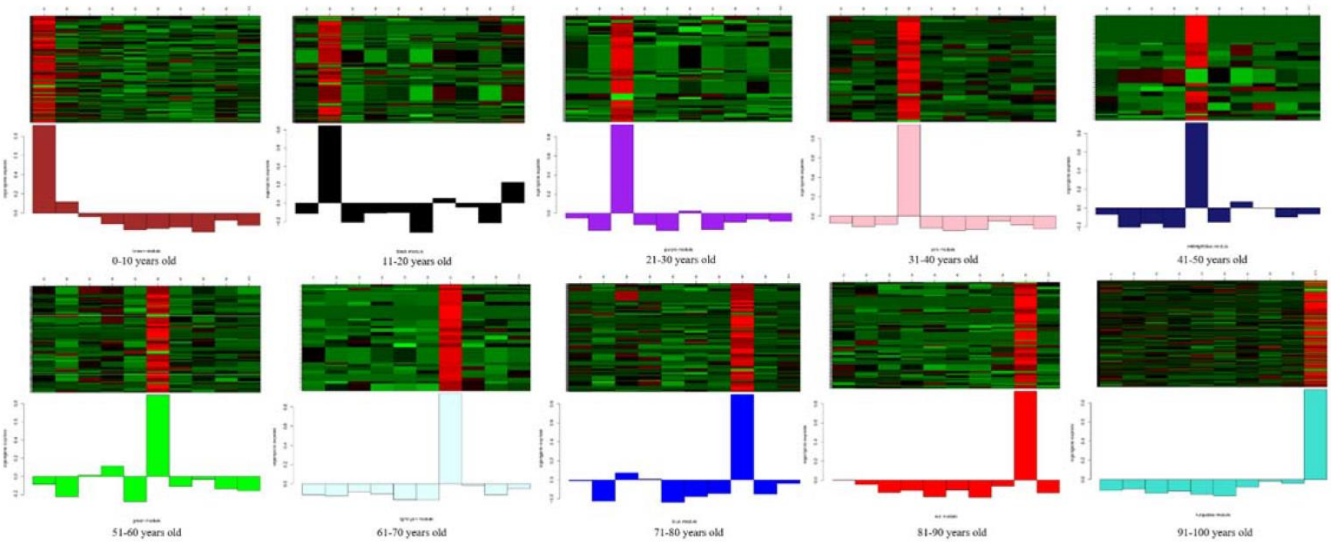

Supplementary Figure 5. The proteins in each module associated with age.

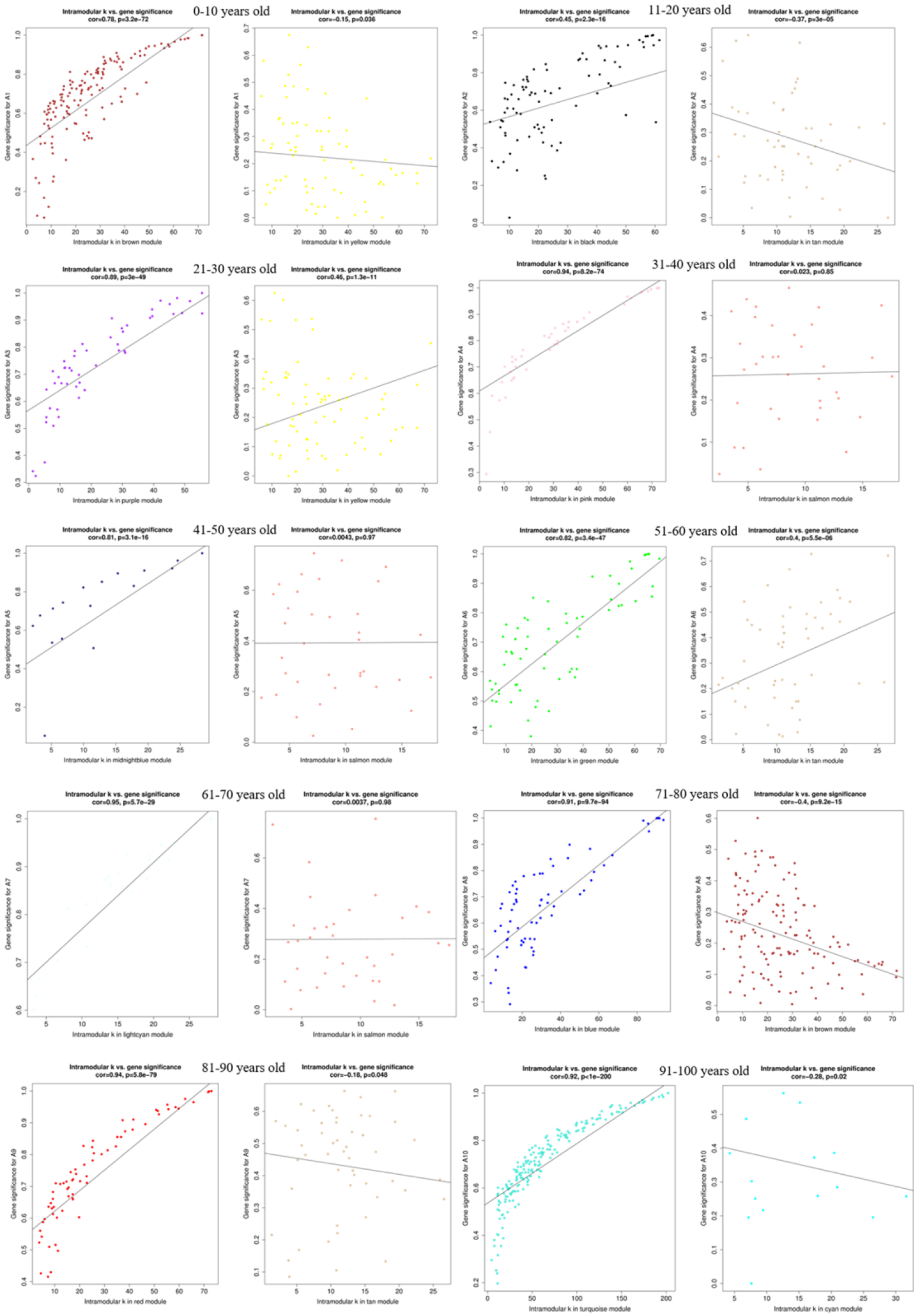

Supplementary Figure 6. Intramodular connectivity (K.in) scatter plot analysis.

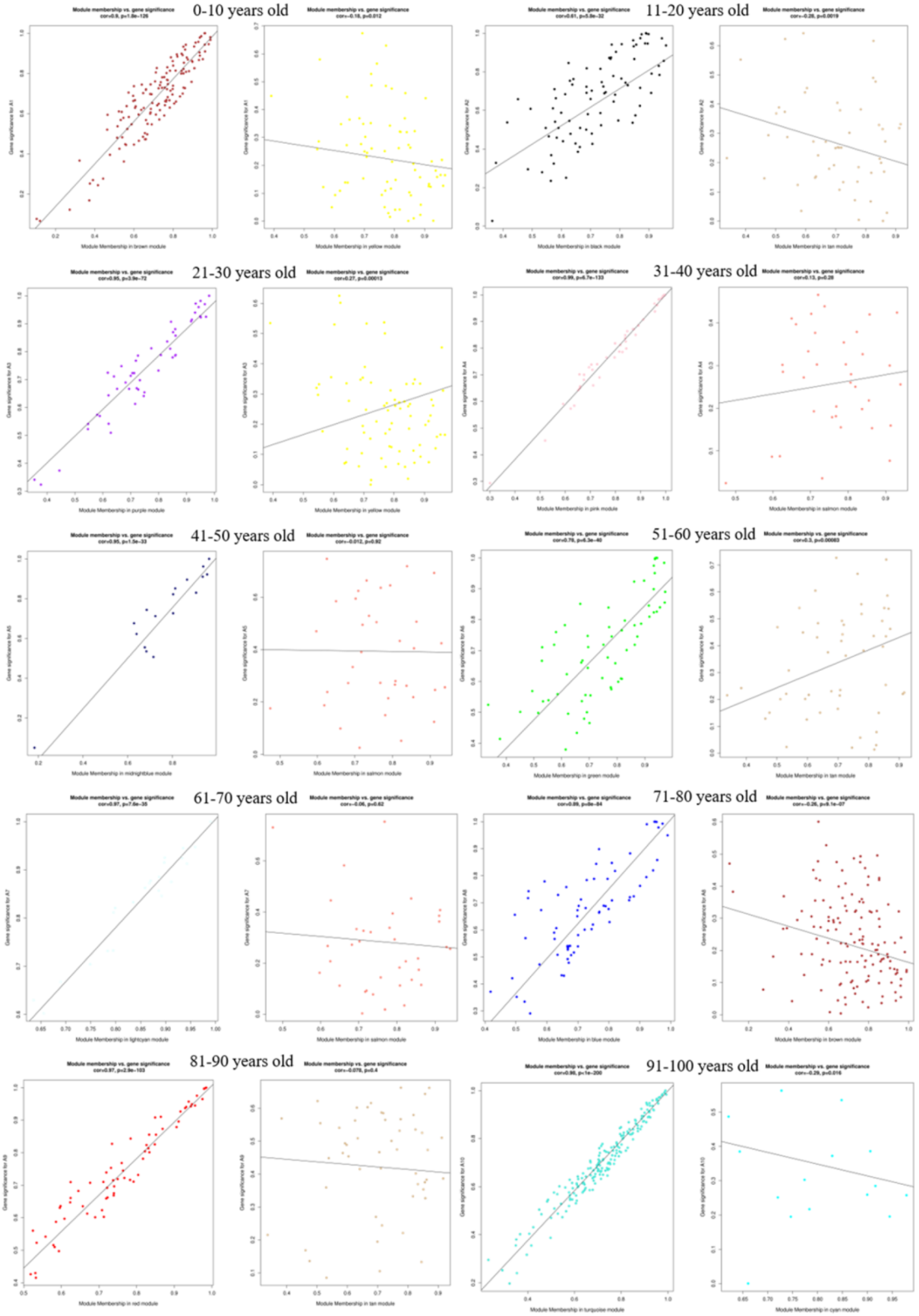

Supplementary Figure 7. Module correlation degree (MM) scatter plot analysis.

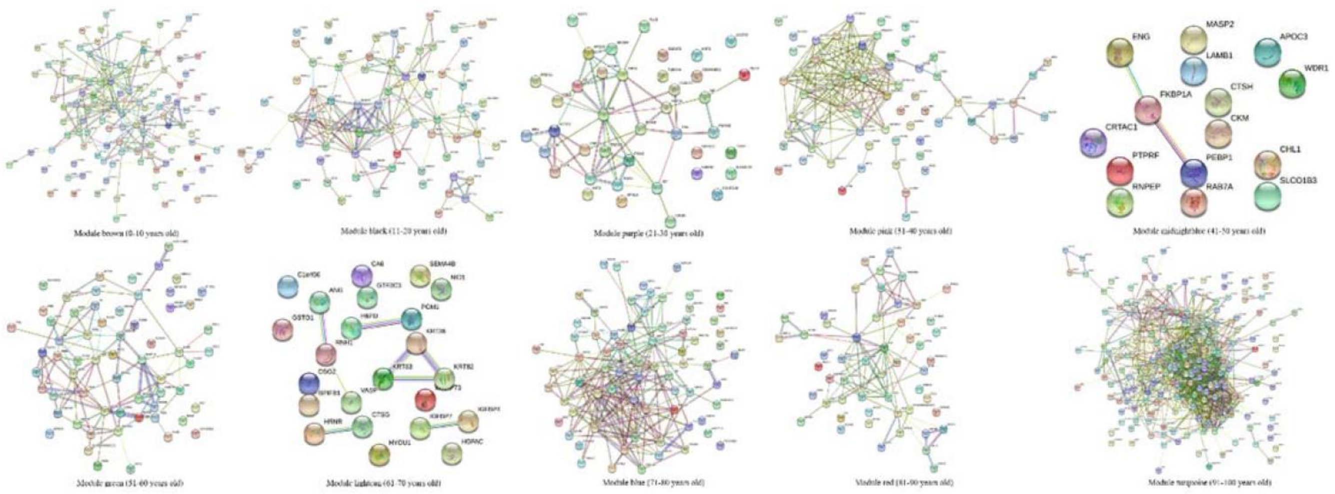

Supplementary Figure 8. Interaction network of proteins in each module associated with age.
