# Supplementary material for: The general law of plasma proteome alterations occurring in the lifetime of Chinese individuals reveals the importance of immunity
Source: Aging (Albany NY). 2022 Sep 7;14(17):7065–92. doi: 10.18632/aging.204278 (PMC9512505; doi:10.18632/aging.204278)
Supplement: Supplementary Tables 1-3 [file aging-14-204278-s002.pdf]

SUPPLEMENTARY TABLES

Supplementary Table 1. Comparisons of gender and ethnicity among the ten groups.

Age	N(Male)	N(Female)	p-value	N(Han)	N(other ethnic)	p-value
0-10	3	6	ref	6	3	ref
11-20	4	6	0.764	9	1	0.213
21-30	1	7	0.312	7	1	0.312
31-40	3	7	0.876	10	0	0.050
41-50	5	5	0.463	9	1	0.213
51-60	4	6	0.764	9	1	0.213
61-70	6	4	0.245	10	0	0.050
71-80	3	7	0.876	10	0	0.050
81-90	4	6	0.764	10	0	0.050
91-100	4	6	0.764	7	3	0.876

N, number.

Supplementary Table 2. The results of protein quantification.

Sample ID	Protein concentration (µg/µl)	Total protein (mg)
1	62.623	3.131
2	111.776	5.589
3	105.119	5.256
4	67.816	3.391
5	111.663	5.583
6	83.133	4.157
7	91.109	4.555
8	105.540	5.277
9	98.105	4.905
10	134.748	6.737
11	110.275	5.514
12	99.361	4.968
13	143.660	7.183
14	89.162	4.458
15	104.221	5.211
16	119.205	5.960
17	159.810	7.991
18	99.511	4.976
19	173.747	8.687
20	148.049	7.402
21	128.456	6.423
22	108.749	5.437
23	171.154	8.558
24	108.014	5.401
25	97.740	4.887
26	85.325	4.266
27	112.743	5.637
28	116.442	5.822
29	106.432	5.322
30	119.538	5.977
31	109.427	5.471
32	141.895	7.095
33	113.742	5.687
34	109.340	5.467
35	97.615	4.881
36	74.705	3.735
37	88.691	4.435
38	100.196	5.010
39	136.595	6.830
40	102.940	5.147
41	101.672	5.084
42	73.901	3.695
43	114.163	5.708
44	134.968	6.748
45	122.829	6.141
46	86.951	4.348
47	92.723	4.636
48	103.895	5.195

49	126.013	6.301
50	158.675	7.934
51	119.344	5.967
52	146.266	7.313
53	116.405	5.820
54	139.000	6.950
55	74.762	3.738
56	112.655	5.633
57	109.314	5.466
58	80.715	4.036
59	99.329	4.966
60	83.026	4.151
61	105.816	5.291
62	111.406	5.570
63	96.352	4.818
64	101.527	5.076
65	90.682	4.534
66	76.018	3.801
67	104.730	5.237
68	102.639	5.132
69	113.491	5.675
70	116.668	5.833
71	110.997	5.550
72	87.077	4.354
73	81.048	4.052
74	75.559	3.778
75	79.635	3.982
76	61.982	3.099
77	74.655	3.733
78	104.843	5.242
79	114.841	5.742
80	85.155	4.258
81	74.454	3.723
82	121.014	6.051
83	134.215	6.711
84	83.836	4.192
85	81.682	4.084
86	106.815	5.341
87	121.322	6.066
88	112.856	5.643
89	90.845	4.542
90	85.846	4.292
91	67.194	3.360
92	96.962	4.848
93	76.357	3.818
94	100.792	5.040
95	96.698	4.835
96	88.389	4.419
97	74.517	3.726

Supplementary Table 3. The top 5 of Gene Ontology (GO)-biological process (BP) in each age group.

Age groups (modules)	GO ID	Description	p-value
	GO:0006956	complement activation	2.245E-57
	GO:0072376	protein activation cascade	2.754E-56
0-10 years old (brown)	GO:0002455	humoral immune response mediated by circulating immunoglobulin	2.522E-53
	GO:0006958	complement activation, classical pathway	2.556E-53
	GO:0006959	humoral immune response	1.125E-49
	GO:0002443	leukocyte mediated immunity	2.597E-14
	GO:0006955	immune response	3.232E-14
11-20 years old (black)	GO:0001775	cell activation	1.282E-13
	GO:0045055	regulated exocytosis	1.478E-13
	GO:0002252	immune effector process	1.539E-13
	GO:0006334	nucleosome assembly	4.122E-23
	GO:0031497	chromatin assembly	6.807E-22
21-30 years old (purple)	GO:0034728	nucleosome organization	4.172E-21
	GO:0006333	chromatin assembly or disassembly	9.201E-21
	GO:0006323	DNA packaging	6.817E-20
	GO:0031424	keratinization	4.207E-28
	GO:0030216	keratinocyte differentiation	5.435E-25
31-40 years old (pink)	GO:0008544	epidermis development	3.925E-23
	GO:0009913	epidermal cell differentiation	3.926E-23
	GO:0043588	skin development	5.422E-23
	GO:1900121	negative regulation of receptor binding	1.135E-04
	GO:1900120	regulation of receptor binding	3.487E-04
41-50 years old (midnightblue)	GO:0061384	heart trabecula morphogenesis	5.989E-04
	GO:0044092	negative regulation of molecular function	1.029E-03
	GO:0010987	negative regulation of high-density lipoprotein particle clearance	1.072E-03
	GO:0045055	regulated exocytosis	9.516E-15
	GO:0006887	exocytosis	1.909E-14
51-60 years old (green)	GO:0016192	vesicle-mediated transport	2.294E-13
	GO:0043312	neutrophil degranulation	2.993E-11
	GO:0002283	neutrophil activation involved in immune response	3.281E-11
	GO:0031424	keratinization	1.636E-05
	GO:0070268	cornification	2.032E-05
61-70 years old (lightcyan)	GO:0030216	keratinocyte differentiation	7.042E-05
	GO:0009913	epidermal cell differentiation	1.684E-04
	GO:0006959	humoral immune response	2.080E-04
	GO:0002576	platelet degranulation	7.913E-16
	GO:0030168	platelet activation	4.977E-13
71-80 years old (blue)	GO:0045055	regulated exocytosis	1.144E-12
	GO:0016192	vesicle-mediated transport	1.335E-12
	GO:0001775	cell activation	1.828E-12
	GO:0072376	protein activation cascade	3.909E-21
	GO:0006956	complement activation	4.606E-19
81-90 years old (red)	GO:0002455	humoral immune response mediated by circulating immunoglobulin	1.445E-18
	GO:0002250	adaptive immune response	4.720E-18
	GO:0006958	complement activation, classical pathway	1.330E-17
	GO:0006342	chromatin silencing	9.295E-51
	GO:0045814	negative regulation of gene expression, epigenetic	1.863E-48
91-100 years old (turquoise)	GO:0000183	chromatin silencing at rDNA	6.490E-46
	GO:0002376	immune system process	1.392E-43
	GO:0006335	DNA replication-dependent nucleosome assembly	9.825E-39
