# Supplementary material for: The general law of plasma proteome alterations occurring in the lifetime of Chinese individuals reveals the importance of immunity
Source: Aging (Albany NY). 2022 Sep 7;14(17):7065–92. doi: 10.18632/aging.204278 (PMC9512505; doi:10.18632/aging.204278)
Supplement: Supplementary Tables 4 and 5 [file aging-14-204278-s003.docx]

**Supplementary Table 4.** The top 10 of Kyoto Encyclopedia of Genes and Genomes (KEGG) in each age group.

| **Age groups (modules)** | **KEGG_A_class** | **KEGG_B_class** | **Pathway** | **p-value** |
| --- | --- | --- | --- | --- |
| 0-10 years old (brown) | Human Diseases | Infectious diseases | Staphylococcus aureus infection | 9.981E-22 |
|  | Cellular Processes | Transport and catabolism | Phagosome | 2.269E-16 |
|  | Human Diseases | Immune diseases | Allograft rejection | 3.058E-16 |
|  | Human Diseases | Cardiovascular diseases | Viral myocarditis | 3.603E-16 |
|  | Human Diseases | Immune diseases | Primary immunodeficiency | 6.604E-16 |
|  | Organismal Systems | Immune system | Intestinal immune network for IgA production | 1.826E-15 |
|  | Organismal Systems | Immune system | Fc gamma R-mediated phagocytosis | 1.879E-15 |
|  | Human Diseases | Immune diseases | Asthma | 2.720E-15 |
|  | Human Diseases | Immune diseases | Autoimmune thyroid disease | 2.769E-15 |
|  | Human Diseases | Infectious diseases | Leishmaniasis | 2.829E-15 |
| 11-20 years old (black) | Environmental Information Processing | Signaling molecules and interaction | ECM-receptor interaction | 4.117E-05 |
|  | Organismal Systems | Immune system | Complement and coagulation cascades | 4.117E-05 |
|  | Human Diseases | Infectious diseases | Staphylococcus aureus infection | 2.051E-04 |
|  | Organismal Systems | Immune system | Antigen processing and presentation | 0.002 |
|  | Human Diseases | Infectious diseases | Tuberculosis | 0.002 |
|  | Organismal Systems | Immune system | Platelet activation | 0.002 |
|  | Cellular Processes | Cellular community - eukaryotes | Focal adhesion | 0.003 |
|  | Metabolism | Carbohydrate metabolism | Amino sugar and nucleotide sugar metabolism | 0.006 |
|  | Cellular Processes | Transport and catabolism | Phagosome | 0.006 |
|  | Human Diseases | Infectious diseases | Amoebiasis | 0.008 |
| 21-30 years old (purple) | Human Diseases | Immune diseases | Systemic lupus erythematosus | 1.102E-24 |
|  | Human Diseases | Cancers | Viral carcinogenesis | 2.193E-20 |
|  | Human Diseases | Substance dependence | Alcoholism | 1.593E-19 |
|  | Organismal Systems | Immune system | Hematopoietic cell lineage | 4.663E-05 |
|  | Cellular Processes | Transport and catabolism | Phagosome | 2.771E-04 |
|  | Human Diseases | Cardiovascular diseases | Dilated cardiomyopathy (DCM) | 3.365E-04 |
|  | Environmental Information Processing | Signaling molecules and interaction | ECM-receptor interaction | 0.002 |
|  | Human Diseases | Cancers | Small cell lung cancer | 0.002 |
|  | Environmental Information Processing | Signal transduction | PI3K-Akt signaling pathway | 0.003 |
|  | Cellular Processes | Cellular community - eukaryotes | Focal adhesion | 0.006 |
| 31-40 years old (pink) | Organismal Systems | Development | Osteoclast differentiation | 8.474E-05 |
|  | Organismal Systems | Immune system | B cell receptor signaling pathway | 1.694E-04 |
|  | Human Diseases | Cardiovascular diseases | Dilated cardiomyopathy (DCM) | 0.020 |
|  | Human Diseases | Infectious diseases | Staphylococcus aureus infection | 0.022 |
|  | Human Diseases | Infectious diseases | Amoebiasis | 0.023 |
|  | Human Diseases | Cardiovascular diseases | Arrhythmogenic right ventricular cardiomyopathy (ARVC) | 0.031 |
|  | Human Diseases | Immune diseases | Systemic lupus erythematosus | 0.039 |
|  | Organismal Systems | Circulatory system | Cardiac muscle contraction | 0.040 |
|  | Environmental Information Processing | Signaling molecules and interaction | ECM-receptor interaction | 0.040 |
|  | Human Diseases | Cardiovascular diseases | Hypertrophic cardiomyopathy (HCM) | 0.041 |
| 41-50 years old (midnightblue) | Human Diseases | Infectious diseases | Amoebiasis | 0.001 |
|  | Organismal Systems | Digestive system | Bile secretion | 0.003 |
|  | Human Diseases | Infectious diseases | Staphylococcus aureus infection | 0.017 |
|  | Cellular Processes | Transport and catabolism | Phagosome | 0.030 |
|  | Human Diseases | Infectious diseases | Tuberculosis | 0.036 |
|  | Metabolism | Amino acid metabolism | Arginine and proline metabolism | 0.061 |
|  | Organismal Systems | Digestive system | Cholesterol metabolism | 0.063 |
|  | Cellular Processes | Transport and catabolism | Mitophagy - animal | 0.079 |
|  | Cellular Processes | Cellular community - eukaryotes | Adherens junction | 0.083 |
|  | Organismal Systems | Endocrine system | PPAR signaling pathway | 0.092 |
| 51-60 years old (green) | Metabolism | Carbohydrate metabolism | Pentose phosphate pathway | 1.764E-05 |
|  | Cellular Processes | Transport and catabolism | Phagosome | 1.908E-05 |
|  | Cellular Processes | Transport and catabolism | Endocytosis | 3.016E-04 |
|  | Metabolism | Carbohydrate metabolism | Glycolysis / Gluconeogenesis | 4.687E-04 |
|  | Cellular Processes | Cellular community - eukaryotes | Focal adhesion | 5.947E-04 |
|  | Cellular Processes | Cell motility | Regulation of actin cytoskeleton | 8.826E-04 |
|  | Human Diseases | Infectious diseases | Pathogenic Escherichia coli infection | 0.003 |
|  | Metabolism | Global and overview maps | Carbon metabolism | 0.004 |
|  | Human Diseases | Infectious diseases | Bacterial invasion of epithelial cells | 0.007 |
|  | Human Diseases | Cardiovascular diseases | Dilated cardiomyopathy (DCM) | 0.011 |
| 61-70 years old (lightcyan) | Metabolism | Carbohydrate metabolism | Pentose phosphate pathway | 0.001 |
|  | Organismal Systems | Immune system | Fc gamma R-mediated phagocytosis | 0.002 |
|  | Human Diseases | Infectious diseases | Staphylococcus aureus infection | 0.002 |
|  | Human Diseases | Infectious diseases | Amoebiasis | 0.002 |
|  | Human Diseases | Immune diseases | Systemic lupus erythematosus | 0.004 |
|  | Human Diseases | Immune diseases | Asthma | 0.011 |
|  | Human Diseases | Infectious diseases | African trypanosomiasis | 0.012 |
|  | Human Diseases | Immune diseases | Allograft rejection | 0.013 |
|  | Human Diseases | Immune diseases | Primary immunodeficiency | 0.014 |
|  | Organismal Systems | Immune system | Intestinal immune network for IgA production | 0.015 |
| 71-80 years old (blue) | Human Diseases | Infectious diseases | Shigellosis | 1.853E-08 |
|  | Cellular Processes | Cell motility | Regulation of actin cytoskeleton | 2.560E-05 |
|  | Cellular Processes | Cellular community - eukaryotes | Focal adhesion | 1.014E-04 |
|  | Cellular Processes | Cellular community - eukaryotes | Adherens junction | 1.597E-04 |
|  | Human Diseases | Cardiovascular diseases | Dilated cardiomyopathy (DCM) | 1.904E-04 |
|  | Organismal Systems | Immune system | Platelet activation | 3.264E-04 |
|  | Organismal Systems | Endocrine system | Relaxin signaling pathway | 3.842E-04 |
|  | Human Diseases | Cardiovascular diseases | Hypertrophic cardiomyopathy (HCM) | 5.283E-04 |
|  | Human Diseases | Infectious diseases | Pathogenic Escherichia coli infection | 9.388E-04 |
|  | Environmental Information Processing | Signal transduction | PI3K-Akt signaling pathway | 0.001 |
| 81-90 years old (red) | Human Diseases | Infectious diseases | Staphylococcus aureus infection | 1.515E-11 |
|  | Human Diseases | Infectious diseases | African trypanosomiasis | 1.470E-09 |
|  | Human Diseases | Immune diseases | Systemic lupus erythematosus | 3.536E-09 |
|  | Human Diseases | Infectious diseases | Amoebiasis | 7.237E-09 |
|  | Human Diseases | Immune diseases | Asthma | 2.722E-08 |
|  | Human Diseases | Immune diseases | Allograft rejection | 4.221E-08 |
|  | Human Diseases | Immune diseases | Primary immunodeficiency | 5.970E-08 |
|  | Organismal Systems | Immune system | Intestinal immune network for IgA production | 9.447E-08 |
|  | Human Diseases | Immune diseases | Autoimmune thyroid disease | 1.140E-07 |
|  | Human Diseases | Cardiovascular diseases | Viral myocarditis | 1.736E-07 |
| 91-100 years old (turquoise) | Human Diseases | Immune diseases | Systemic lupus erythematosus | 1.605E-60 |
|  | Human Diseases | Substance dependence | Alcoholism | 7.476E-41 |
|  | Human Diseases | Cancers | Transcriptional misregulation in cancers | 3.955E-16 |
|  | Organismal Systems | Immune system | Complement and coagulation cascades | 1.399E-15 |
|  | Human Diseases | Infectious diseases | Staphylococcus aureus infection | 9.679E-14 |
|  | Human Diseases | Infectious diseases | African trypanosomiasis | 6.230E-11 |
|  | Cellular Processes | Cell growth and death | Necroptosis | 5.777E-08 |
|  | Organismal Systems | Immune system | Intestinal immune network for IgA production | 2.263E-07 |
|  | Human Diseases | Cardiovascular diseases | Viral myocarditis | 5.991E-07 |
|  | Human Diseases | Immune diseases | Primary immunodeficiency | 7.459E-07 |

**Supplementary Table 5.** The protein–protein interaction (PPI) analysis with high combined scores (value > 0.9) and low co-expression coefficients (value = 0) in different modules.

| Age groups | module | node1 | node2 | node1_connectivity | node2_connectivity | coexpression | combined_score |
| --- | --- | --- | --- | --- | --- | --- | --- |
| 0-10 years old | brown | A2M | THBS1 | 71.308 | 32.630 | 0 | 0.919 |
|  |  | A2M | PF4 | 71.308 | 22.782 | 0 | 0.917 |
|  |  | A2M | RAC1 | 71.308 | 9.440 | 0 | 0.901 |
|  |  | ALB | THBS1 | 19.905 | 32.630 | 0 | 0.942 |
|  |  | ALB | VCAN | 19.905 | 13.500 | 0 | 0.935 |
|  |  | ALB | PF4 | 19.905 | 22.782 | 0 | 0.942 |
|  |  | AP2A1 | APOB | 67.125 | 24.428 | 0 | 0.902 |
|  |  | AP2A2 | APOB | 67.125 | 24.428 | 0 | 0.902 |
|  |  | APOB | PPP2R1A | 24.428 | 49.231 | 0 | 0.902 |
|  |  | APOB | VCAN | 24.428 | 13.500 | 0 | 0.915 |
|  |  | C2 | IGHV3-11 | 10.516 | 38.729 | 0 | 0.916 |
|  |  | C3 | TUBB | 10.587 | 26.672 | 0 | 0.901 |
|  |  | C3 | GDI2 | 10.587 | 24.081 | 0 | 0.905 |
|  |  | C3 | VCAN | 10.587 | 13.500 | 0 | 0.905 |
|  |  | C3 | CFHR4 | 10.587 | 16.678 | 0 | 0.986 |
|  |  | CADM1 | RAC1 | 27.511 | 9.440 | 0 | 0.906 |
|  |  | CDH5 | RAC1 | 42.155 | 9.440 | 0 | 0.956 |
|  |  | FLNA | GP1BA | 32.067 | 15.967 | 0 | 0.997 |
|  |  | IGHV3-11 | IGLL1 | 38.729 | 17.640 | 0 | 0.903 |
|  |  | IGHV3-11 | RAC1 | 38.729 | 9.440 | 0 | 0.909 |
|  |  | ILK | RAC1 | 16.942 | 9.440 | 0 | 0.940 |
|  |  | ISLR | THBS1 | 28.432 | 32.630 | 0 | 0.903 |
|  |  | KRT31 | KRT6A | 53.557 | 7.239 | 0 | 0.917 |
|  |  | MADCAM1 | SELL | 55.054 | 47.163 | 0 | 0.962 |
|  |  | PF4 | SERPINF2 | 22.782 | 27.220 | 0 | 0.952 |
|  |  | PLXNB1 | RAC1 | 7.730 | 9.440 | 0 | 0.955 |
|  |  | SPP2 | VCAN | 85.653 | 13.500 | 0 | 0.901 |
| 11-20 years old | black | APOH | LGALS3BP | 12.410 | 40.441 | 0 | 0.905 |
|  |  | CPNE3 | VNN1 | 22.850 | 24.960 | 0 | 0.906 |
|  |  | HLA-C | LILRB5 | 54.932 | 14.197 | 0 | 0.918 |
|  |  | HSP90AA1 | RHOA | 9.581 | 43.932 | 0 | 0.936 |
|  |  | PROS1 | TGFB1 | 25.466 | 23.316 | 0 | 0.908 |
|  |  | RHOA | UBB | 43.932 | 54.320 | 0 | 0.943 |
|  |  | TGFB1 | UBC | 23.316 | 54.320 | 0 | 0.913 |
|  |  | TGFB1 | UBB | 23.316 | 54.320 | 0 | 0.915 |
|  |  | TGFB1 | VWF | 23.316 | 19.263 | 0 | 0.935 |
| 21-30 years old | purple | FN1 | ITGA2B | 2.220 | 13.710 | 0 | 0.974 |
|  |  | IGFBP5 | P4HB | 47.543 | 16.061 | 0 | 0.912 |
| 31-40 years old | pink | APCS | ITM2B | 13.282 | 16.991 | 0 | 0.906 |
|  |  | ATP2A2 | ATP2A3 | 58.830 | 58.830 | 0 | 0.991 |
| 41-50 years old | midnightblue | ENG | FKBP1A | 24.610 | 6.653 | 0 | 0.905 |
| 51-60 years old | green | CANX | HLA-B | 53.815 | 29.770 | 0 | 0.931 |
|  |  | CAP1 | ORM2 | 60.382 | 3.816 | 0 | 0.903 |
|  |  | DBNL | HP | 13.671 | 9.353 | 0 | 0.902 |
|  |  | HP | LTA4H | 9.353 | 58.221 | 0 | 0.902 |
|  |  | ORM2 | TUBB4B | 3.816 | 49.988 | 0 | 0.901 |
|  |  | TPM1 | TPM3 | 50.490 | 18.609 | 0 | 0.986 |
|  |  | TUBB1 | TUBB4B | 55.006 | 49.988 | 0 | 0.901 |
| 61-70 years old | lightcan | ANG | RNH1 | 13.201 | 19.421 | 0 | 0.994 |
| 71-80 years old | blue | ACTA1 | CAPNS1 | 57.476 | 12.953 | 0 | 0.913 |
|  |  | ACTA1 | ACTB | 57.476 | 25.966 | 0 | 0.945 |
|  |  | ACTN1 | FERMT3 | 26.473 | 20.679 | 0 | 0.922 |
|  |  | ACTN1 | PPBP | 26.473 | 12.052 | 0 | 0.907 |
|  |  | ALDOA | PPBP | 19.708 | 12.052 | 0 | 0.904 |
|  |  | ALDOA | TMSB4X | 19.708 | 22.000 | 0 | 0.901 |
|  |  | APP | F13A1 | 90.890 | 14.639 | 0 | 0.901 |
|  |  | APP | PPBP | 90.890 | 12.052 | 0 | 0.904 |
|  |  | APP | CCL5 | 90.890 | 90.879 | 0 | 0.921 |
|  |  | ARF1 | IGF2R | 24.929 | 91.610 | 0 | 0.938 |
|  |  | CTSD | PPBP | 90.651 | 12.052 | 0 | 0.904 |
|  |  | CYB5R3 | UNC13D | 19.525 | 17.090 | 0 | 0.901 |
|  |  | CYB5R3 | GGH | 19.525 | 12.453 | 0 | 0.902 |
|  |  | GGH | VCL | 12.453 | 62.691 | 0 | 0.901 |
|  |  | GNB1 | GNB4 | 36.576 | 36.577 | 0 | 0.901 |
|  |  | GNB1 | PPBP | 36.576 | 12.052 | 0 | 0.904 |
|  |  | GNB2 | GNB4 | 36.576 | 36.577 | 0 | 0.901 |
|  |  | HSPA8 | IGF2R | 29.567 | 91.610 | 0 | 0.917 |
|  |  | HSPA8 | TF | 29.567 | 25.781 | 0 | 0.913 |
|  |  | IGF2R | TF | 91.610 | 25.781 | 0 | 0.904 |
|  |  | LGALS1 | TF | 33.306 | 25.781 | 0 | 0.901 |
|  |  | PPBP | TMSB4X | 12.052 | 22.000 | 0 | 0.924 |
| 81-90 years old | red | A1BG | FN1 | 9.290 | 2.220 | 0 | 0.917 |
|  |  | FN1 | SERPINA10 | 2.220 | 20.239 | 0 | 0.903 |
| 91-100 years old | turquoise | AHSG | APOL1 | 72.648 | 49.616 | 0 | 0.912 |
|  |  | AHSG | MMRN1 | 72.648 | 12.623 | 0 | 0.957 |
|  |  | AHSG | CST3 | 72.648 | 106.644 | 0 | 0.944 |
|  |  | APOA1 | HSPG2 | 48.668 | 25.556 | 0 | 0.915 |
|  |  | APOA1 | LRP1 | 48.668 | 33.877 | 0 | 0.919 |
|  |  | APOA1 | PLTP | 48.668 | 43.537 | 0 | 0.946 |
|  |  | APOA1 | APOL1 | 48.668 | 49.616 | 0 | 0.982 |
|  |  | APOA2 | CETP | 48.962 | 29.800 | 0 | 0.923 |
|  |  | APOA2 | LRP1 | 48.962 | 33.877 | 0 | 0.910 |
|  |  | APOA2 | APOL1 | 48.962 | 49.616 | 0 | 0.949 |
|  |  | APOA2 | HSPG2 | 48.962 | 25.556 | 0 | 0.912 |
|  |  | APOA2 | CST3 | 48.962 | 106.644 | 0 | 0.926 |
|  |  | APOA2 | PLTP | 48.962 | 43.537 | 0 | 0.943 |
|  |  | APOE | QSOX1 | 20.459 | 42.256 | 0 | 0.902 |
|  |  | APOE | APOL1 | 20.459 | 49.616 | 0 | 0.949 |
|  |  | APOL1 | SERPIND1 | 49.616 | 53.829 | 0 | 0.917 |
|  |  | APOL1 | ITIH2 | 49.616 | 132.353 | 0 | 0.904 |
|  |  | APOL1 | SERPINC1 | 49.616 | 58.881 | 0 | 0.912 |
|  |  | APOL1 | TF | 49.616 | 25.781 | 0 | 0.912 |
|  |  | APOL1 | HBB | 49.616 | 35.110 | 0 | 0.921 |
|  |  | APOL1 | CST3 | 49.616 | 106.644 | 0 | 0.932 |
|  |  | C3 | MPO | 10.587 | 185.840 | 0 | 0.951 |
|  |  | C3 | PRDX6 | 10.587 | 22.160 | 0 | 0.909 |
|  |  | CAMP | CST3 | 139.437 | 106.644 | 0 | 0.907 |
|  |  | CDH1 | PROC | 49.940 | 32.936 | 0 | 0.913 |
|  |  | CDH1 | IGF2 | 49.940 | 75.265 | 0 | 0.943 |
|  |  | CDH1 | PLG | 49.940 | 91.386 | 0 | 0.948 |
|  |  | CDH1 | IGF1 | 49.940 | 24.699 | 0 | 0.971 |
|  |  | CETP | PON1 | 29.800 | 44.433 | 0 | 0.913 |
|  |  | CFD | FGB | 88.190 | 45.765 | 0 | 0.916 |
|  |  | CFP | TIMP2 | 18.840 | 33.178 | 0 | 0.901 |
|  |  | CFP | LCN2 | 18.840 | 196.683 | 0 | 0.903 |
|  |  | CLU | IGF1 | 32.568 | 24.699 | 0 | 0.964 |
|  |  | CLU | MMRN1 | 32.568 | 12.623 | 0 | 0.916 |
|  |  | CLU | QSOX1 | 32.568 | 42.256 | 0 | 0.925 |
|  |  | COLEC11 | FCN3 | 85.887 | 24.488 | 0 | 0.973 |
|  |  | CST3 | PROC | 106.644 | 32.936 | 0 | 0.907 |
|  |  | CST3 | FGA | 106.644 | 45.072 | 0 | 0.913 |
|  |  | CST3 | FGG | 106.644 | 48.344 | 0 | 0.903 |
|  |  | CST3 | HP | 106.644 | 9.353 | 0 | 0.941 |
|  |  | CST3 | ITIH2 | 106.644 | 132.353 | 0 | 0.908 |
|  |  | CST3 | SERPINC1 | 106.644 | 58.881 | 0 | 0.923 |
|  |  | CST3 | HSPG2 | 106.644 | 25.556 | 0 | 0.908 |
|  |  | ELANE | ERP44 | 28.866 | 46.771 | 0 | 0.901 |
|  |  | ELANE | TIMP2 | 28.866 | 33.178 | 0 | 0.928 |
|  |  | ELANE | PRDX6 | 28.866 | 22.160 | 0 | 0.930 |
|  |  | ELANE | QSOX1 | 28.866 | 42.256 | 0 | 0.903 |
|  |  | ELANE | NPC2 | 28.866 | 139.321 | 0 | 0.902 |
|  |  | ERP44 | HP | 46.771 | 9.353 | 0 | 0.901 |
|  |  | F2 | SLC9A3R1 | 50.781 | 57.549 | 0 | 0.917 |
|  |  | FABP5 | PRDX6 | 48.701 | 22.160 | 0 | 0.908 |
|  |  | FCGR3B | GPLD1 | 12.596 | 40.580 | 0 | 0.902 |
|  |  | FCN3 | MASP1 | 24.488 | 8.840 | 0 | 0.981 |
|  |  | FGB | MMRN1 | 45.765 | 12.623 | 0 | 0.915 |
|  |  | HBB | LRP1 | 35.110 | 33.877 | 0 | 0.901 |
|  |  | HBB | TIMP2 | 35.110 | 33.178 | 0 | 0.902 |
|  |  | HBB | HBE1 | 35.110 | 24.150 | 0 | 0.902 |
|  |  | HP | TIMP2 | 9.353 | 33.178 | 0 | 0.910 |
|  |  | HP | OSCAR | 9.353 | 77.826 | 0 | 0.901 |
|  |  | HRG | QSOX1 | 52.422 | 42.256 | 0 | 0.910 |
|  |  | HRG | MMRN1 | 52.422 | 12.623 | 0 | 0.912 |
|  |  | HSPG2 | TTR | 25.556 | 69.847 | 0 | 0.946 |
|  |  | HSPG2 | PLG | 25.556 | 91.386 | 0 | 0.932 |
|  |  | IGF1 | QSOX1 | 24.699 | 42.256 | 0 | 0.903 |
|  |  | IGF2 | QSOX1 | 75.265 | 42.256 | 0 | 0.902 |
|  |  | IGF2 | IGFALS | 75.265 | 57.615 | 0 | 0.944 |
|  |  | MMRN1 | PLG | 12.623 | 91.386 | 0 | 0.908 |
|  |  | MPO | TTR | 185.840 | 69.847 | 0 | 0.921 |
|  |  | MPO | PRDX6 | 185.840 | 22.160 | 0 | 0.924 |
|  |  | NPC2 | PRTN3 | 139.321 | 108.508 | 0 | 0.904 |
|  |  | NPC2 | TTR | 139.321 | 69.847 | 0 | 0.921 |
|  |  | PRDX6 | PRTN3 | 22.160 | 108.508 | 0 | 0.905 |
|  |  | PRDX6 | TTR | 22.160 | 69.847 | 0 | 0.910 |
|  |  | SOD1 | SOD3 | 27.524 | 46.105 | 0 | 0.914 |
|  |  | TF | TFRC | 25.781 | 14.064 | 0 | 0.994 |
