# Supplementary material for: The general law of plasma proteome alterations occurring in the lifetime of Chinese individuals reveals the importance of immunity
Source: Aging (Albany NY). 2022 Sep 7;14(17):7065–92. doi: 10.18632/aging.204278 (PMC9512505; doi:10.18632/aging.204278)
Supplement: Supplementary Table 6 [file aging-14-204278-s004.pdf]

Supplementary Table 6. The molecular functions of hub genes.

Age groups	Module	Hub genes	Connectivity	Description	KEGG class
0-10 years old	brown	ARPC2	16.766	actin related protein 2/3 complex subunit 2	Human Diseases;Cellular Processes; Organismal Systems
		ARPC5	18.201	actin related protein 2/3 complex subunit 5	Human Diseases;Cellular Processes; Organismal Systems
11-20 years old	black	DDX39B	49.029	DExD-box helicase 39B	Genetic Information Processing
		HNRNPK	64.876	heterogeneous nuclear ribonucleoprotein K	Human Diseases; Genetic Information Processing
		HNRNPA2B1	66.056	heterogeneous nuclear ribonucleoprotein A2/B1	-
		EEF2	69.684	eukaryotic translation elongation factor 2	Organismal Systems;Environmental Information Processing
		UBA52	54.320	ubiquitin A-52 residue ribosomal protein fusion product 1	Genetic Information Processing
		RPS27A	54.320	ribosomal protein S27a	Genetic Information Processing
		UBB	54.320	ubiquitin B	Human Diseases;Cellular Processes
		UBC	54.320	ubiquitin C	Organismal Systems
51-60 years old	green	ADH1A	65.236	alcohol dehydrogenase 1A (class I), alpha polypeptide	Metabolism;Human Diseases
		ADH1B	65.236	alcohol dehydrogenase 1B (class I), beta polypeptide	Metabolism;Human Diseases
		ARPC3	22.205	actin related protein 2/3 complex subunit 3	Human Diseases;Cellular Processes;Organismal Systems
		ARPC4	24.819	actin related protein 2/3 complex subunit 4	Human Diseases;Cellular Processes;Organismal Systems
		PGD	8.834	phosphogluconate dehydrogenase	Metabolism
		TALDO1	4.664	transaldolase 1	Metabolism
71-80 years old	blue	ACTB	25.966	actin beta	Human Diseases;Organismal Systems;Cellular Processes;Environmental Information Processing
		ACTC1	57.476	actin alpha cardiac muscle 1	Organismal Systems;Human Diseases
		ACTBL2	52.264	actin beta like 2	-
		ACTC1	57.476	actin alpha cardiac muscle 1	Organismal Systems;Human Diseases
		COL1A1	16.750	collagen type I alpha 1 chain	Human Diseases;Environmental Information Processing;Cellular Processes;Organismal Systems
		COL1A2	90.917	collagen type I alpha 2 chain	Human Diseases; Environmental Information Processing; Cellular Processes; Organismal Systems
81-90 years old	red	CALR	14.716	calreticulin	Human Diseases; Genetic Information Processing; Cellular Processes; Organismal Systems
		HSPA5	13.575	heat shock protein family A (Hsp70) member 5	Genetic Information Processing; Organismal Systems; Human Diseases; Genetic Information Processing
91-100 years old	turquoise	AHSG	72.648	alpha 2-HS glycoprotein	-
		APOA1	48.668	apolipoprotein A1	Organismal Systems; Human Diseases
		FGA	45.072	fibrinogen alpha chain	Organismal Systems
		F2	50.781	coagulation factor II, thrombin	Human Diseases; Environmental Information Processing; Cellular Processes; Organismal Systems
		FGG	48.344	fibrinogen gamma chain	Organismal Systems; Human Diseases
		ITIH2	132.353	inter-alpha-trypsin inhibitor heavy chain 2	-
		SERPINC1	58.881	serpin family C member 1	Organismal Systems
		APOA2	48.962	apolipoprotein A2	Organismal Systems
		APOM	28.989	apolipoprotein M	-
		FGB	45.765	fibrinogen beta chain	Organismal Systems
		C1QB	28.621	complement C1q B chain	Human Diseases; Organismal Systems

VSIG4	11.071	V-set and immunoglobulin domain containing 4	Organismal Systems
EEF1B2	46.912	eukaryotic translation elongation factor 1 beta 2	-
F2	50.781	coagulation factor II, thrombin	Human Diseases; Environmental Information Processing; Cellular Processes; Organismal Systems
TPT1	202.100	tumor protein, translationally-controlled 1	-
FETUB	19.827	fetuin B	-
